# A human corticospinal organoid-slice connectoid model informs enhancer strategies for post-injury axon regrowth

**DOI:** 10.1016/j.celrep.2026.117399

**Published:** 2026-05-26

**Authors:** George M. Gibbons, Tanja Fuchsberger, Mai Abdelgawad, Stefano L. Giandomenico, Kornélia Szebényi, Veselina Petrova, Lea M.D. Wenger, Daniel N. Olschewski, Jeremi Chabros, Leila Muresan, Rachael C. Feord, Muhammad Asif, James W. Fawcett, Susanna B. Mierau, Ole Paulsen, Madeline A. Lancaster, András Lakatos

**Affiliations:** 1 John van Geest Centre for Brain Repair, Department of Clinical Neurosciences, University of Cambridge, Cambridge, UK; 2 Department of Physiology, Development and Neuroscience, University of Cambridge, Cambridge, UK; 3 MRC Laboratory of Molecular Biology, Cambridge, UK; 4 Division of Cognitive and Behavioral Neurology, Brigham & Women’s Hospital, Boston, MA, USA; 5 MRC-WT Cambridge Stem Cell Institute, Cambridge Biomedical Campus, Cambridge, UK

## Abstract

Axon elongation in the mammalian central nervous system (CNS) declines during development, limiting regenerative capacity after birth. Intrinsic regulators of this process are promising repair targets, as immature axons can regrow in tissues otherwise not conducive to regeneration. Yet the precise timing and mechanisms underlying the cessation of axon growth in the human CNS remain unresolved. Here, we developed a three-dimensional human corticospinal motor organoid-slice connectoid platform mimicking the developmental axon elongation program and its subsequent restriction through maturation. Cortical and spinal slices establish functional connections while remaining spatially segregated, enabling cortical cell-type-specific observations without direct confounding effects by spinal cells. Using single-cell transcriptomics, computational analyses, axon regrowth assays, and live imaging, we identified transcriptional alterations contributing to decreased axon growth in maturing human cortical projection neurons. We further demonstrate that this decline can be reversed using compounds and repurposable drugs targeting a maturation-associated transcriptional shift, promoting post-injury axon repair.

## Graphical abstract

**Graphical abstract:**
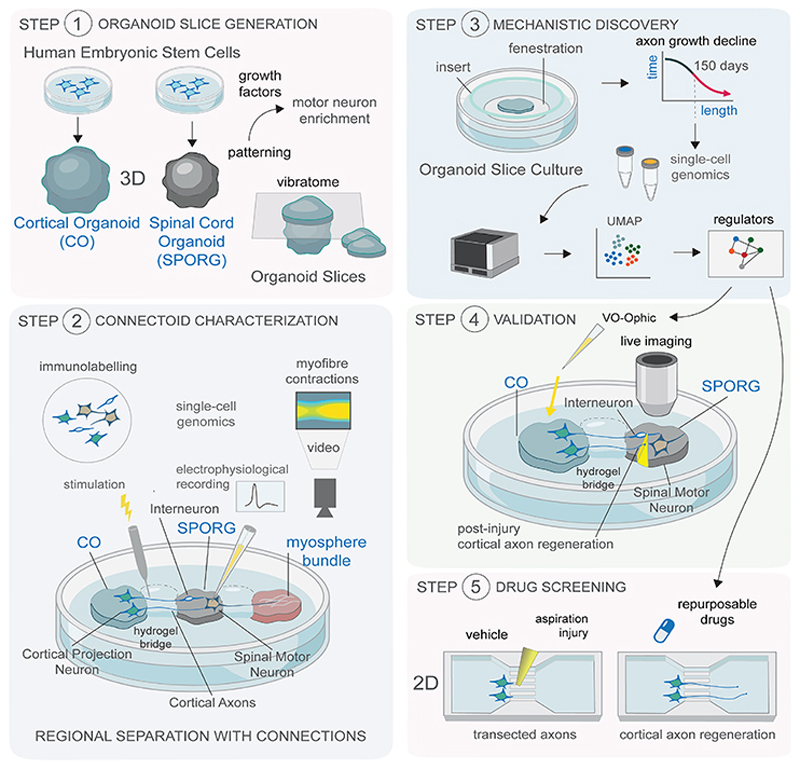

## Introduction

While spinal cord regeneration is preserved throughout the life-time of several vertebrate species, including amphibians, limited repair underlies the permanent motor disabilities in humans following central nervous system (CNS) damage.^1,2^ Several intrinsic neuronal and extrinsic restrictive causes have been proposed, increasing the complexity of targeting strategies.^3,4^ The ability of immature neurons to extend their axons in CNS environments characterized by extracellular molecules that are non-permissive for repair steers the focus toward intrinsic drivers of axon elongation.^5,6^ This ability declines while neurons mature and is significantly attenuated at birth,^6,7^ which is transcriptionally regulated in rodent models,^6,8^ providing cues for a potential targeting approach.^9^ However, whether such developmental restriction occurs in the human CNS remains unresolved. The therapeutically relevant translation of findings has been hampered not only by the increasingly recognized interspecies cellular differences but also by the lack of high-fidelity systems mimicking cell-type diversity, cell interactions, and tissue architecture of the human brain and spinal cord.

Three-dimensional patient-specific induced pluripotent stem cell (iPSC)-derived organoids emerge as vital discovery models shedding light on human aspects of neural physiology and disease.^10^ The assembly of organoid systems, in which axons of CTIP2-expressing cortical projection neurons are connected to spinal circuits with output to muscle fibers, has begun.^11,12^ Nevertheless, multiregional assembloids, in which organoids are fused together, remain somewhat restricted in their ability to model distinct tissue environments, in which specific cell types can be analyzed without a direct confounding effect exerted by cells of a different region. Crucially, complex organoid systems have not been utilized to elucidate the causes and treatment options for human corticospinal axon repair failure associated with injury or degeneration.

To overcome these barriers, we generated and validated a human corticospinal connectoid system, comprising regionally segregated air-liquid interface cortical and spinal organoid slice cultures, while possessing functional synapses and motor output to myocyte spheres (myospheres). The organoid slice-based platform provided longevity for cell maturation and connections through improved nutrition,^11^ as well as high accessibility to a flat surface for consistent positioning, molecular manipulations, and imaging. We show that our human developmental model mimics the late-fetal and postnatal restriction of axon regeneration in mature corticospinal neurons, which contributes to repair failure in spinal cord injury (SCI) since early life. By subjecting the cortical organoid slice and connectoid discovery platform to single-cell RNA sequencing (scRNA-seq) analyses and axon regrowth assays combined with live imaging and perturbations, we uncover transcriptional changes and mechanisms underlying the developmental decline in axon elongation in human cortical projection neurons. Furthermore, we demonstrate that we can overcome the growth blockade by targeting the drivers of this process, highlighting the translational value of our system. Utilizing an *in silico* drug pre-selection method and mid-throughput assays, we demonstrate that repurposable medications with a potential to reverse cortical projection neuron maturation-related transcriptional changes can enhance injury-evoked human cortical axon regrowth.

## Results

### Anterior spinal cord cell-type diversity is recapitulated in organoids

**Figure 1:**
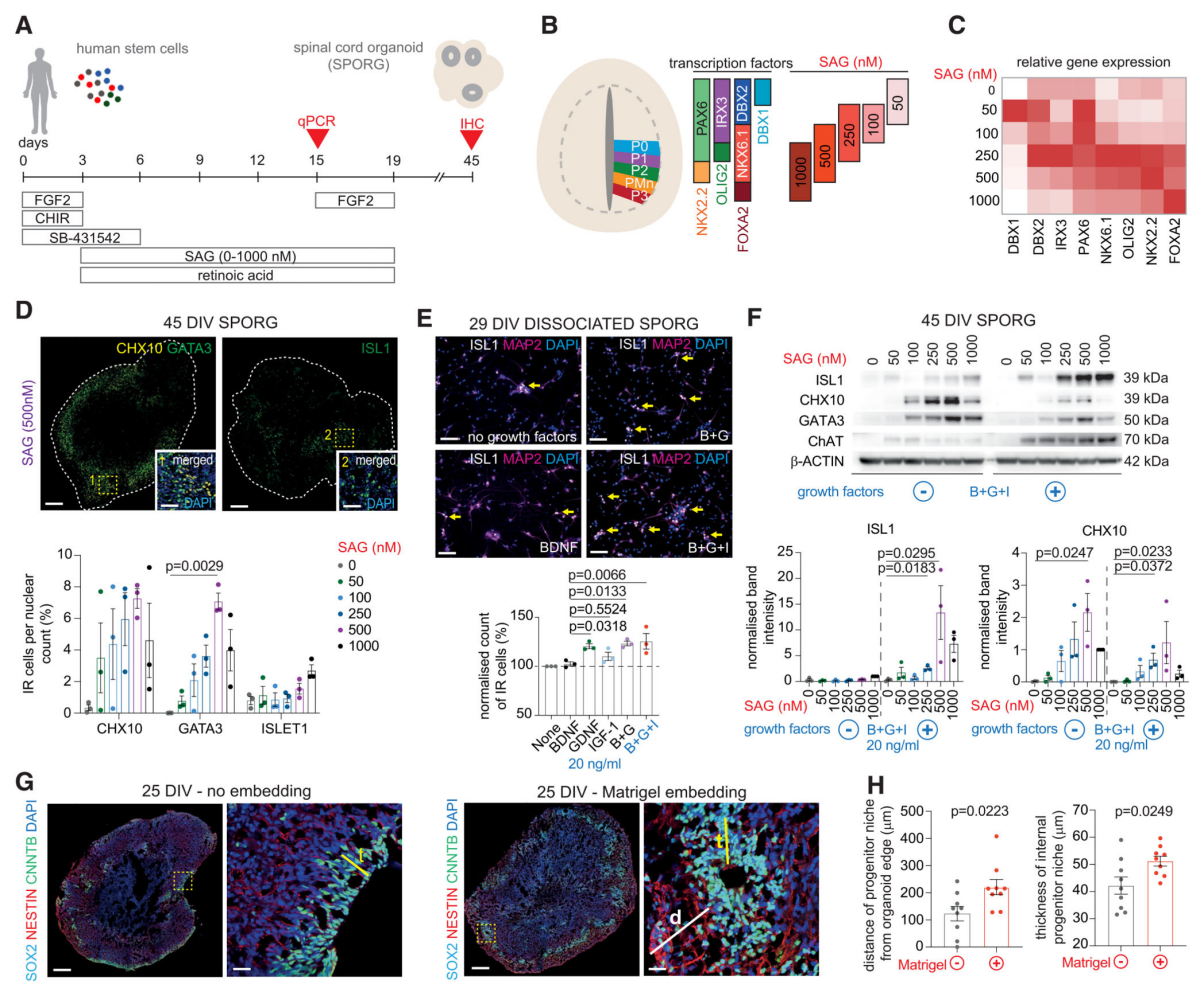
Spinal cord organoid cell-type diversity and architecture are optimized by SAG, growth factors, and embedding (A) Schematic of spinal cord organoid (SPORG) generation. (B) Schematic illustrating anterior spinal cord domains in development (left) and the domain-specific transcription factors (TFs) expression in SPORGs treated with increasing smoothened agonist (SAG), a Sonic Hedgehog analog. (C) Heatmap of SAG-induced relative expression of domain-specific TFs. (D) Representative stitched tile-scan immunofluorescence (IF) images (top) illustrating CHX10^+^ V2a interneurons (INs), GATA3^+^ V2b INs, and ISL1^+^ motor neurons (MNs). Insets show merged images with DAPI staining at 45 days *in vitro* (DIV). Graph (bottom) demonstrates mean ± SEM percentage of immunoreactive (IR) cells per DAPI count in SPORGs treated with SAG. *N* = 3 independent batches of SPORGs (3 per batch), Kruskal-Wallis test with Dunn’s multiple comparisons. Scale bars: 200 and 60 μm (inset). (E) Representative merged IF images (top) showing ISL1^+^/MAP2^+^ MNs dissociated and cultured from SPORGs at 15 DIV and untreated or treated with BDNF, BDNF+GDNF (B + G), and BDNF+GDNF+IGF-1 (B + G + I) for 14 days at 20 ng/mL concentration. Graphs (bottom) show the mean ± SEM ISL1^+^ MN counts in cultures treated with growth factors, normalized to untreated cultures. *N* = 3 independent cell cultures of dissociated SPORGs, one-way ANOVA with Dunnett’s multiple comparisons to BDNF treatment. Scale bars: 70 μm. (F) Representative western blots for CHX10, GATA3, and ISL1 with β-ACTIN (top) of 45 DIV SPORG samples either untreated or treated with increasing concentrations of SAG and the B + G + I GF cocktail. Graphs (bottom) demonstrating the mean ± SEM IR band densities normalized to the corresponding β-ACTIN^+^ bands and to ISL1^+^ or CHX10^+^ bands for the 1,000 nM SAG-treated SPORG samples in the same blot. *N* = 3 independent batches of SPORGs (3 per batch), Kruskal-Wallis test with Dunn’s multiple comparisons. (G) Representative stitched tile-scan IF images of non-embedded and Matrigel-embedded SPORGs treated with 500 nM SAG, showing SOX2^+^, NESTIN^+^, and CNNTB^+^ cells in progenitor niche areas at 25 DIV. Lines represent niche distance (d) from the organoid edge and niche thickness (t). Scale bars: 200 and 20 μm (insets). (H) Graphs represent mean ± SEM of progenitor niche distance from the organoid edge and niche thickness in non-embedded and Matrigel-embedded SPORGs at 25 DIV. *N* = 9 independent SPORG cultures, two-tailed unpaired *t* test. See also Figure S1.

To generate a human corticospinal motor organoid-slice connectoid with defined cortical and spinal cord tissue regions, we first optimized spinal cord organoid (SPORG) cultures grown from a stable pluripotent H9 human embryonic stem cell (hESC) line for anterior positional identity and motor neuron (MN) enrichment (Figures S1A and S1B). The initial step, similar to a published protocol,^13^ involved patterning using a varying concentration (0 and 1,000 nM) of smoothened agonist (SAG), a small-molecule activator of the Sonic Hedgehog (SHH) signaling pathway, in addition to caudalization using retinoic acid (Figure 1A). This resulted in a relatively high expression of anterior domain transcription factors (TFs) in SPORGs at 250–500 nM of SAG, including NKX6.1 and OLIG2, as demonstrated by qPCR at 15 days *in vitro* (DIV) (Figures 1B, 1C, and S1C). To tailor cell-type composition and architecture in SPORGs for motor circuits, we determined which SAG concentration enriches crucial cell types innervated by corticospinal neurons in humans. Immunophenotyping at 45 DIV indicated that the treatment with 500 nM of SAG leads to the highest proportion of CHX10^+^ V2a excitatory and GATA3^+^ inhibitory V2b interneurons (INs; Figures 1D and S1D). At the same time, the density of ISL1^+^ MNs remained relatively low (Figure 1D). Since MN survival is heavily dependent on particular growth factors (GFs), we tested whether, at this SAG concentration, we can increase their abundance by administering combinations of BDNF, GDNF, and insulin growth factor (IGF).^13–15^ For a rapid assessment, we immunolabeled dissociated cell cultures of SPORGs following *N*-[*N*-(3,5-difluorophenacetyl)-L-alanyl]-*S*-phenylglycine *t*-butyl ester (DAPT)-induced terminal differentiation at 15 DIV.^16^ The proportion of ISL1^+^ MNs was increased by 20.29% and 22.44% in response to BDNF+GDNF (B + G) and BDNF+GDNF+IGF (B + G + I) when compared to BDNF alone 2 Cell Reports *45*, 117399, June 23, 2026 (Figure 1E). The abundance of B + G + I-induced MNs was corroborated by an increase in band densities of ISL1 protein levels in immunoblots, which was the greatest at 41.92-fold when SPORG-derived cultures were co-treated with 500 nM of SAG. Notably, CHX10^+^ and GATA3^+^ band intensities, reflecting V2a and V2b IN presence, remained the highest at 250–500 nM of SAG treatment, irrespective of B + G + I administration (Figures 1F and S1E). Despite recapitulating spinal cord cell types, the germinal layers in the SPORGs showed inverse polarity, situated at the tissue edge (Figure 1G). Thus, we explored if the fidelity of tissue organization can be increased by embedding in Matrigel, serving as polarizing factors that mimic basal lamina.^17,18^ Progenitor zones in embedded SPORGs were significantly more distant from the edge, and their thickness was 1.21 times more extensive than that of non-embedded organoids (Figures 1G and 1H). The density of germinal areas was comparable (Figure S1F). These findings indicate that the combination of SAG (250–500 nM), B +G + I GFs, and embedding (SGE treatment onward) can optimize anterior motor domain patterning in SPORGs (Figure S1G).

### SGE treatment induces glial and neuronal specification in SPORG slice cultures

**Figure 2:**
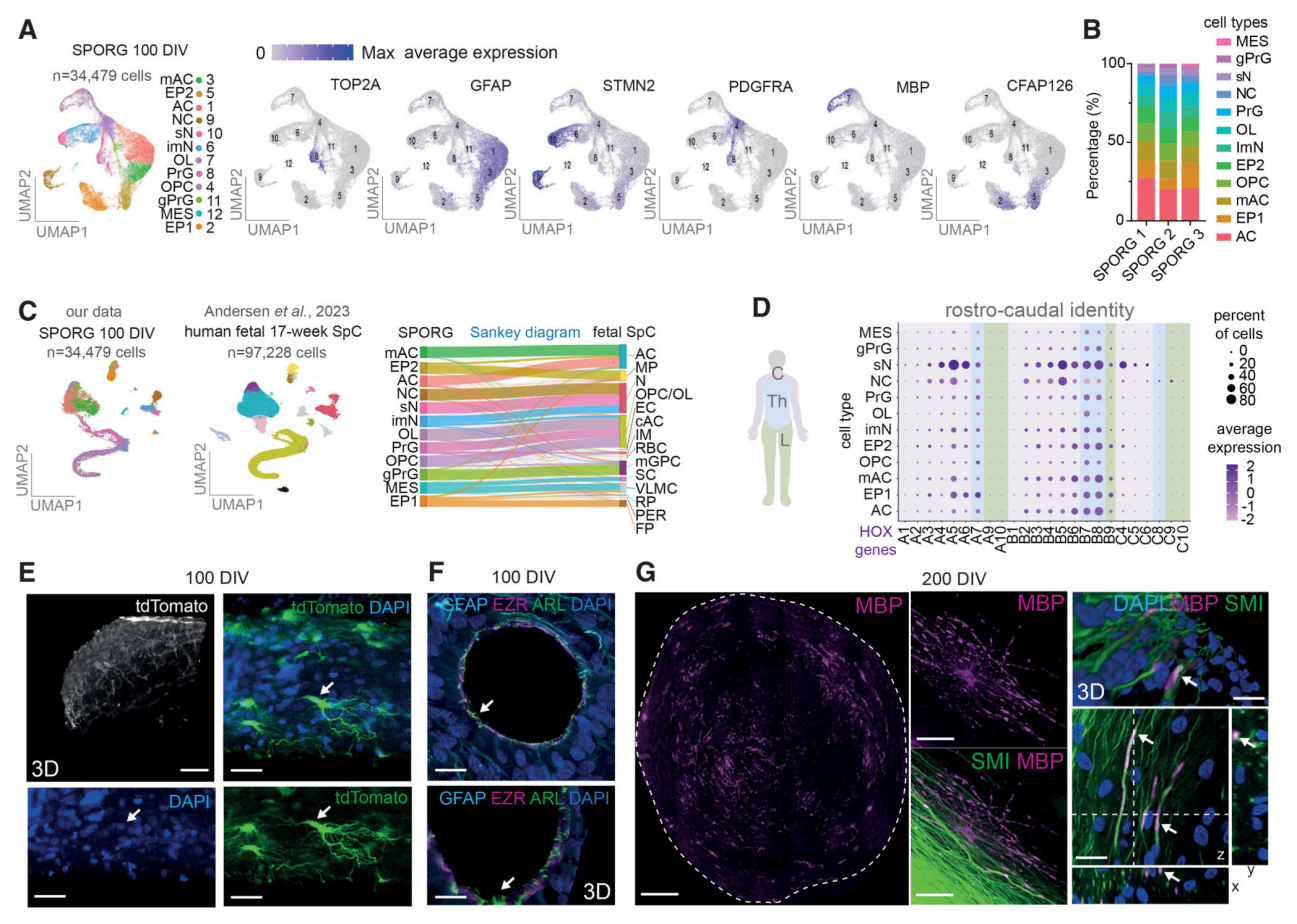
Spinal cord organoids comprised transcriptionally and morphologically mature cell types (A) UMAP (left) and feature maps (right) representing diverse cell types (color coding) and marker gene expression in various clusters (numbers), respectively, in merged single-cell RNA sequencing (scRNA-seq) datasets of spinal cord organoids (SPORGs) at 100 days *in vitro* (DIV). Color scale shows the log-normalized expression values from zero to the highest value (Max). AC, astroglia; EP, ependymal cell; mAC, mature astrocyte; OPC, oligodendrocyte progenitor cell; imN, immature neuron; OL, oligodendrocyte; PrG, progenitor; NC, neural stem cell; sN, spinal neuron; gPrG, glial progenitor cells; MES, mesenchymal cell. *N* = 34,479 cells from 3 independent SPORG cultures. (B) Stacked bar chart showing the percentage of cell types of all cells per SPORG in the scRNA-seq datasets. (C) UMAPs representing the scRNA-seq SPORG dataset (left) and a published dataset of 17-week-old (119 days) human fetal spinal cord (SpC) samples (middle) in the same UMAP space with overlapping cell-type profiles, corroborated by the strength of correlations and visualized in a Sankey diagram. Greater connection thickness (variance flow) indicates higher correlations between SPORG and human fetal spinal cord cells. (D) Dot plots showing the percentage (dot size) of HOX gene-expressing cells and the average expression level (color coding), indicating rostrocaudal cell identities at 100 DIV in SPORGs. (E) Representative 3D (top left) and stack of confocal microscopy images of SPORGs, demonstrating RFP immunoreactive (IR) AAV-GFAP-tdTomato-transduced astroglial cells and/or DAPI^+^ nuclei at 100 DIV. Scale bars: 100 μm (3D), 40 μm. (F) Representative 3D (bottom) and stack of confocal microscopy images of SPORGs, showing a central canal-like formation demarcated by ARL^+^/EZRIN^+^ microvilli of endothelial cells intermingling with GFAP^+^ glial processes at 100 DIV. Scale bars: 15 and 25 μm (3D). (G) Representative stacks of stitched tile-scan confocal microscopy images of SPORGs, illustrating MBP+ oligodendrocytes and enwrapped SMI^+^ neurites (arrows). 3D images (top right) and orthogonal planes (bottom right) show myelin-neuron contacts at 200 DIV. Scale bars: 500 μm (left), 50 μm (middle), 20 μm (right). See also Figure S2.

We then tested whether the SGE treatment enables the maturation of motor circuit-forming neurons in SPORG slice cultures at 100 DIV. For this, SPORGs were treated with 375 nM SAG, a concentration falling between those that yielded abundant MNs while maintaining INs. Furthermore, we adapted our previously published organoid slice culture method to SPORGs, thereby improving nutrition, longevity, and accessibility.^11^ This enabled single-cell transcriptomics as well as morphological and electrophysiological assessments. Using the 10× Chromium scRNA-seq platform, reads were aligned in CellRanger, and further data processing was performed in Seurat using our standard protocols for sliced organoids.^19^ The merged dataset included transcripts from 34,479 cells from three independent SPORG slices, resulting in 12 unbiased clusters (Figure 2A). These clusters were annotated for progenitors and several mature glial, neuronal, and ependymal cell types, showing consistent cell population proportions across three biological repeats of SPORG samples (Figures 2A and 2B). Merging our SPORG dataset with a human fetal spinal cord dataset^20^ in the same uniform manifold approximation and projection (UMAP) space showed overlapping cell-type profiles. To define this using a metric, we generated shared nearest neighbor (SNN) profiles, providing connecting edges between SPORG and fetal cell types, and calculated the fraction of all connections to a specific cell type to provide a similarity score (0–1). We then visualized the strength of relationships in a Sankey diagram, as connecting strips with varying thickness from SPORG cell types to their fetal cell counterparts, which corroborated transcriptional profile overlaps for each major cell population (Figure 2C). Moreover, the HOX gene-based regional identity analysis indicated that SGE-treated SPORG slices possessed a broad rostro-caudal signature, with prominent cervical and thoracic transcriptomic and protein marker profiles (HOXA3-A7, HOXB2-B8, and HOXC4-C8) in addition to thoracolumbar gene signatures (HOXB9 and HOXC9), which were less prominent in other studies using assembloids^12^ (Figures 2D and S2A).

To explore cues for cell maturity and potential functional specification, cell subpopulations were analyzed by immunolabeling and differentially expressed genes (DEGs) within subclustered scRNA-seq datasets. Differentiated astrocytes displayed a typical process arborization visualized using an AAV5-GFAP-tdTomato vector and RFP immunolabeling (Figure 2E). The transcriptional profile of astroglial lineage cells indicated the presence of radial glia (*FABP7*), immature astroglia (*HES6*), and *AQP4*/*GJA1*-expressing differentiated astrocytes with a potential impact on synaptogenesis (*SPARCL1*) (Figures 2E, S2B, and S2C). Cells with DAPI-stained nuclei were distributed in typical palisades juxtaposed by GFAP^+^ glial processes around a lumen-like structure, mimicking the architecture of the central canal (Figure 2F). These cells were identified as ependymal cells, based on acetylated tubulin (AC-TUB)-AQP4 co-labeling and ARL13B^+^/EZRIN^+^ cilia (Figures 2F and S2D). Transcriptionally, they showed a phenotypic separation into anterior (*ARX*, 4 Cell Reports *45*, 117399, June 23, 2026 *SULF*1, and *FOXA1*) and lateral (*PAX6*) domain-like populations (Figure S2E). The anterior axon guidance molecule-expressing floor plate-like ependyma (e.g., *EFNB3*-expressing cells) consisted of *NTN1*- and non-*NTN1*-expressing cells (Figure S2F), potentially identifying them as populations that signpost commissural axons during brain development.^21^ One lateral domain-like population displayed gene-expression similarities to GFAP-expressing astroglial cells, enriched in synaptogenic genes (*GPC3* and *SPARCL1*), indicating their potential developmental links to the early astroglial lineage (Figure S2F). The other lateral cluster indicated enrichment in cytoskeletal and cilia-related gene expression (*ROPN1L, DAAM1*, and *C11ORF88*), reflecting a subpopulation with a role in cerebrospinal fluid circulation^22^ (Figure S2F). SGE-treated SPORGs also comprised mature-looking oligodendrocytes with a bona fide arborized morphology. They displayed widespread MBP^+^ myelin immunoreactivity adjacent to neurofilament (SMI)^+^ axons, which was corroborated by transmission electron microscopy and their gene expression profile (*MAG, MBP, CLDN11, GSN*, and *NKX6.2*) (Figures 2G and S2G–S2I). In addition, the transcriptional signatures also reflected the presence of oligodendrocyte progenitor cells (*PDGFRA*) and pre-myelinating progenitors (*PTPRZ1*) (Figure S2I).

**Figure 3:**
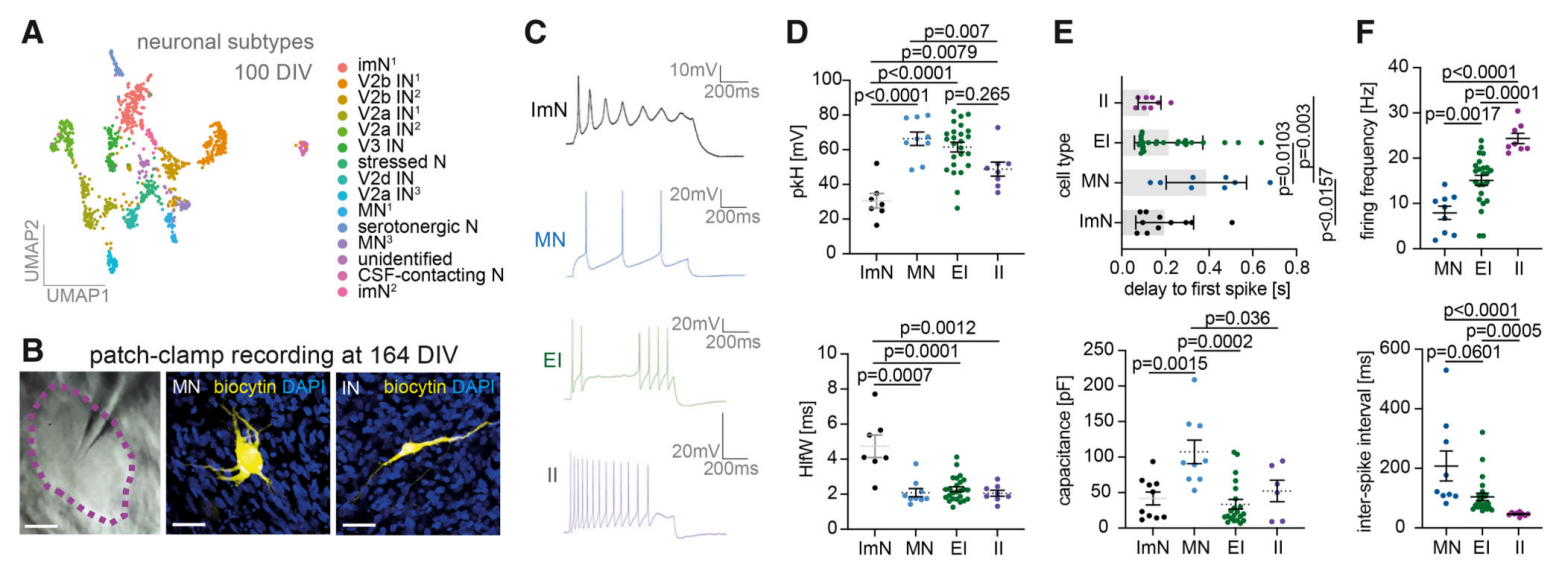
Spinal cord organoids display functionally mature motor neurons and interneurons (A) UMAP representing the isolated neuronal dataset of three independent spinal cord organoids (SPORGS) at 100 days *in vitro* (DIV). imN, immature neuron; IN, interneuron; MN, motor neuron; CSF-contacting N, cerebral spinal fluid-contacting neuron. (B) Whole-cell patch clamping illustrated by differential interference contrast microscopy image of a pipette within a neuron (left) filled with biocytin and co-stained post hoc with DAPI (right) after recording at 164 DIV in SPORGs. Scale bars: 20 μm (left), 70 μm (right, for MN and IN). (C) Representative traces of action potentials (APs) evoked with a step current demonstrating distinct spike and firing properties of an immature neuron (ImN), motor neuron (MN), excitatory interneuron (EI), and inhibitory interneuron (II) from the recording of eight independent SPORG batches. (D) Graphs indicating the mean ± SEM distinct AP peak height (pkH) (top) and half-width (HfW, bottom) in neurons. *N* = 9, 7, 25, and 8 cells (from 8 batches), respectively; unpaired *t* test with Welch’s correction (pkH); Mann-Whitney test (HfW). (E) Graphs show the mean ± SEM delay from the current step onset to the first spike (top) and capacitance of MNs (bottom). *N* = 8, 25, 8, and 11 cells (from 8 batches), respectively (for delay to first spike); Mann-Whitney test; *N* = 10, 9, 21, and 6 cells (from 8 batches), respectively (for capacitance); Mann-Whitney test. (F) Graphs show the mean ± SEM firing frequencies (top) and inter-spike interval characteristics (bottom) in neurons. *N* = 9, 24, and 8 cells (from 8 batches), respectively; one-way ANOVA with Tukey’s multiple comparisons (firing frequency); *N* = 9, 24, and 9 cells (from 8 batches), respectively; Kruskal-Wallis test with Dunn’s multiple comparisons. See also Figure S3.

To assess spinal neuronal populations^23^ in SPORGs at fine-grained resolution, we subclustered the isolated neuronal dataset, which uncovered 15 subtypes, including immature neurons and mature motor circuit-forming cells, in addition to stressed cells and an unidentified population (Figure 3A). The TF analysis in this cell lineage mirrored the temporal sequences of spinal cord cell differentiation from the early stages marked by *ONECUT1,2,3* expression, progressing toward neuronal specification and maturation reflected by transcripts, such as *NFIA, NFIB*, and *NEUROD2* (Figure S3A). We could distinguish immature neurons and two MN types in addition to excitatory and inhibitory IN subtypes, including two V2a and two V2b populations, V2d, V3, and serotonergic INs, supported by their domain marker and neurotransmitter expression profiles (Figures 3A and S3B). The analysis was also suggestive of the presence of cerebral spinal fluid (CSF)-contacting neurons (Figure 3A). To evaluate the electrophysiological maturity of these neurons, wholecell patch-clamp recordings were carried out, and step current injections were used to evoke action potentials (APs) (Figures 3B and S3C–S3H). The analysis of spike train dynamics, AP parameters, and input resistance distinguished immature neurons with small and wide APs from putative excitatory/inhibitory IN and MN profiles demonstrating mature spiking properties (Figure 3C). MNs, with confirmed identity through combined live biocytin labeling and ChAT immunofluorescence, had longer hyperpolarization times, lower firing frequencies, and extended inter-spike intervals than INs (Figures 3B–3F, S3C, and S3D). Our findings offer consistent morphological, transcriptional, and electrophysiological evidence of mature anterior circuit-forming neuronal populations in SPORGs.

### Regionally distinct but connected cortical and spinal organoid slices drive muscle contractions

**Figure 4:**
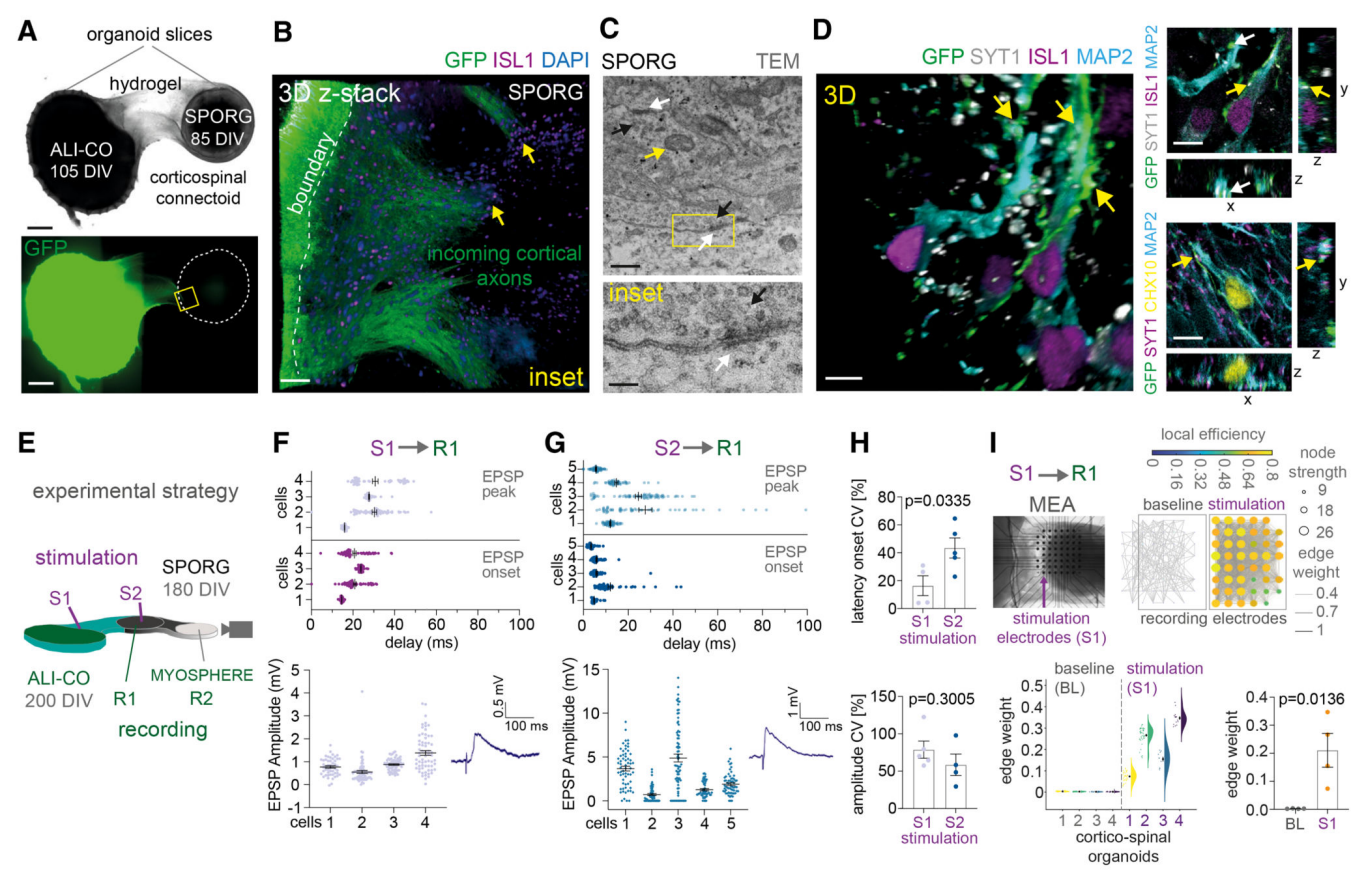
The cortical and spinal organoid slices are connected by functional synapses (A) Representative phase contrast (top) and stitched tile-scan GFP immunofluorescence (IF) image (bottom) showing the human corticospinal motor organoidslice connectoid 35 days after assembled from 70 days *in vitro* (DIV) air-liquid interface cortical organoid slices (ALI-COs) to 50 DIV spinal cord organoid (SPORG) slices on fenestrated culture inserts, showing connections through the hydrogel bridge. Scale bars: 1 mm. (B) Inset demonstrating 3D-reconstructed GFP^+^ immunoreactive (IR) cortical axons, deeply penetrating and projecting onto ISL1^+^ MNs in the SPORG. Scale bars: 50 μm. (C) Transmission electron microscopy (TEM) images of synapses in SPORGs, showing presynaptic vesicles (black arrows) juxtaposed by the postsynaptic density (white arrows), magnified in the inset. Scale bars: 400 and 120 nm (inset). (D) Representative 3D reconstruction (left) and in orthogonal planes (right) of confocal microscopy images, showing SYT1^+^ presynapytic terminals of ALI-CO-derived GFP^+^ axons projecting onto MAP2^+^/ISL1^+^ MNs. Scale bars: 7.5 μm (3D), 10 μm. (E) Schematic of evaluating functional connectivity in the corticospinal organoid-slice connectoid system by electrophysiological single-cell patch-clamp recordings (R1) in SPORGs (180 DIV) following ALI-CO (200 DIV) stimulation (S1, S2; 120 days after organoid assembly). (F) Graphs show the mean ± SEM excitatory postsynaptic potential (EPSP) onset and peak (top) and amplitudes (bottom left) with a representative trace (bottom right) recorded in SPORG neurons following extracellular stimulation of tracts formed by cortical neurons (S1). *N* = 4 neurons with multiple recordings per cell (technical replicates). (G) Graphs demonstrate the mean ± SEM EPSP onset and peak (top) and amplitudes (bottom left) with a representative trace (bottom right) recorded from SPORG neurons following stimulation of local connections in SPORGs (S2). *N* = 5 neurons with multiple recordings per cell. (H) Graphs illustrating the mean ± SEM coefficient of variation (CV) of EPSP onset latencies (top) and amplitudes (bottom) after stimulation at S1 and S2 sites. *N* = 4, 5 and 5, 4 neurons, respectively, with multiple recordings per cell, unpaired two-tailed *t* tests. (I) Phase contrast image (top left) illustrates a corticospinal organoid on the microelectrode array (MEA) with overlaying stimulating electrodes (first column dots, S1) placed on corticospinal tracts (arrow) or recording electrodes (3–8 columns of dots) over the SPORG. Representative network graphs (top right) show functional connectivity within the SPORG at baseline and during corticospinal stimulation. Node strength (circle size), edge weight (line thickness), and local efficiency (node color) indicate neuronal firing rates, connection strength, and domain activity, respectively. Scatterplots indicate mean ± SEM edge weight with density curves for each SPORG (bottom left) and for all SPORGs with bars (bottom right) before and during stimulation. *N* = 4 independent corticospinal organoidslice connectoids with multiple stimulations and recordings per connectoid, two-tailed unpaired *t* test. See also Figure S4.

**Figure 5:**
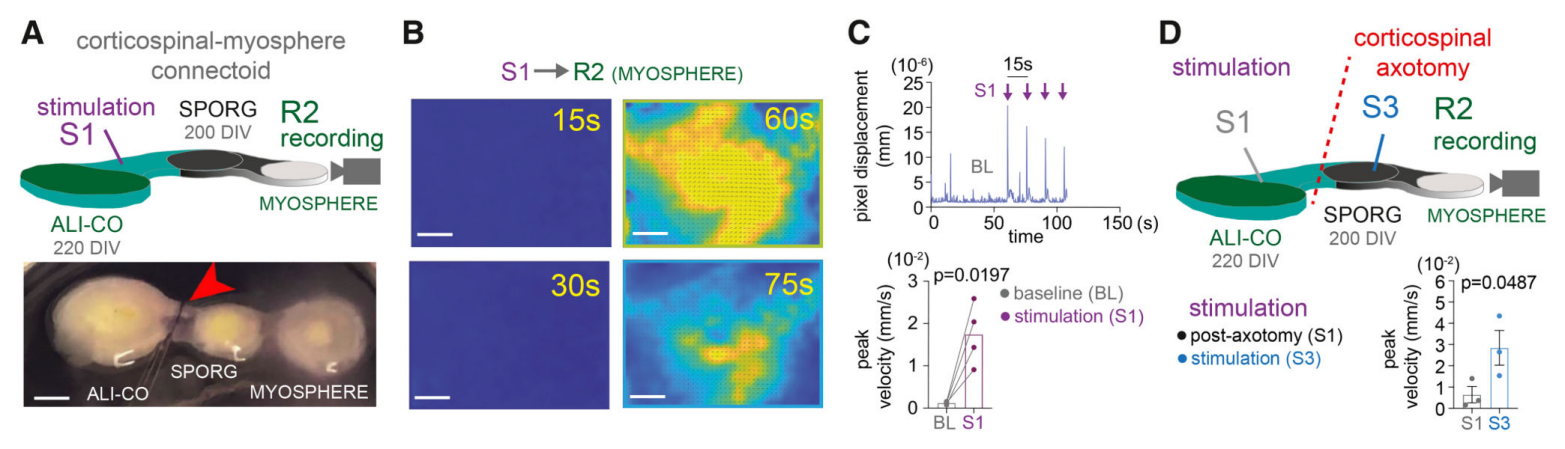
The functionally connected human corticospinal motor organoid neuraxis drives myosphere contractions (A) Schematic (top) and photograph (bottom) of the human corticospinal organoid slice-myosphere connectoid model, indicating video recording (R2) of myosphere contractions following the stimulation (S1) of the outgrowing axon tracts (red arrow) of 220 days *in vitro* (DIV) air-liquid interface cortical organoids (ALI-COs) connected to 200 DIV spinal cord organoids (SPORG) (150 days after assembly). Scale bars: 3 mm. (B) Heatmaps illustrating low (low) and high (yellow) particle velocity readings from video recordings of myosphere contractions before and after cortical organoid stimulation, respectively. Scale bars: 225 μm. (C) Representative example of pixel displacement traces (top) over time with intermittent stimulations (15 s apart). Graph (bottom) shows the paired plots and the mean of first peak velocities (greatest displacement over 1.5 s) in videos of myosphere contractions at baseline and upon stimulation of cortical organoids within the connected corticospinal-myosphere organoid system. *N* = 4 independent connectoids (stimulation repeated 4 times), two-tailed paired *t* test. (D) Schematic of post-axotomy myosphere contraction measurements upon stimulation of the outgrowing ALI-CO axon tracts (S1) or the SPORG (S2). Graph shows mean ± SEM pixel displacement (mm) in videos of myosphere contractions after severing ALI-CO-SPORG connections upon stimulation of outgrowing axons from ALI-COs (S1) and upon direct stimulation of SPORGs (S3). *N* = 3 independent connectoids (stimulation repeated 4 times), unpaired *t* test with Welch’s correction. See also Figure S4.

Next, we addressed whether SPORG neurons can be connected and activated by the outgrowing axons of cortical neurons. For this, we have grown cortical organoid slices at the air-liquid interface (ALI-COs) from the H9-GFP hESC reporter line (Figure 4A), in which the axon outgrowth of CTIP2^+^ deep-layer corticospinal-like neurons was previously demonstrated.^11^ The GFP^+^ ALI-CO axons outgrowing on culture inserts were bridged to SPORGs by 1% alginate/Matrigel (1:1) solution (AMS). Visualized by fluorescence and phase contrast microscopy, these GFP^+^ axons deeply penetrated and reached the adjacent ISL1^+^ SPORGs placed 2 mm apart within 4–8 weeks (Figures 4A and 4B). The ultrastructural evidence of synapses in SPORGs prompted further analyses to determine the specificity of synaptic connections between GFP^+^ ALI-CO axon terminals and SPORG MNs and INs (Figure 4C). We used triple-immunolabeling for pre- and postsynaptic markers, SYT1 and HOMER, and neuron-specific markers, analyzed by confocal microscopy. The proximity of GFP^+^ axon terminals, synaptic puncta, and MAP2^+^ structures of ISL1 or CHX10 immunoreactive (IR) cells suggested connections between corticospinal tracts and SPORG MN and IN neurons, respectively (Figure 4D). To confirm functional corticospinal synaptic input, indicated by evoked excitatory postsynaptic potentials (EPSPs), we conducted whole-cell patch-clamp recordings of neurons located in SPORGs following extracellular stimulation of the outgrowing tracts of cortical neurons or local connections (Figures 4E and S4A). When stimulating the area close to the neuronal cell bodies, the mean onset of EPSPs was 6.4 ± 1.5 ms with peaks at 16.9 ± 4.1 ms, while corticospinal tract stimulations resulted in longer and less variable delays in both EPSP onset (20 ± 1.9 ms) and peak (26.2 ± 3.5 ms) (Figure 4F). The latter, with reduced variability in EPSP delays, indicates monosynaptic corticospinal connections of MNs and excitatory INs, which were also visualized in functional ISL1 and CHX10 IR cells, respectively (Figures 4D and 4F). In contrast, the increased variability in EPSP onset after direct stimulation of SPORG cells suggests relayed MN and IN activation (Figures 4G and 4H). Using microelectrode arrays (MEAs), we then examined whether intraspinal organoid neuronal activity is driven by the stimulation of axons of corticospinal-like neurons. The increase in post-stimulation correlation between the firing patterns of various cortical plate domains during stimulation of the corticospinal tracts, compared with spontaneous activity, provides evidence for excitable neuronal networks in the SPORGs (Figures 4I and S4B). To verify that neuronal activity specifically involves functional motor units, we assembled corticospinal organoid slices with stem cell-derived myospheres, leaving a 2 mm gap bridged by AMS (Figure 5A). Within the corticospinal organoid slice-myosphere connectoid, corticospinal-like tracts extended from ALI-COs were stimulated using current pulses through an electrode (Figure 5A). Then, video-recorded muscle contractions were analyzed using particle-velocity vector maps in time-sequence image stacks (Figure 5B). The sum of velocity vectors was negligible before or between the stimulation of tracts formed by the axons of outgrowing cortical neurons. It peaked at the evoked electrical bursts, illustrated by heatmaps, and the maximum velocity magnitudes were significantly higher upon stimulation (Figures 5B, 5C, and S4C). We then cut the axon tracts between the ALI-CO and SPORG domains to confirm that muscle contractions were the result of conduction via corticospinal axons rather than the potential spread of extracellular currents over the insert surface (Figure 5D). Transections silenced muscle contractions upon stimulation, showing only residual spontaneous activity. However, the synchronous movements were retained upon the direct stimulation of SPORGs (Figure 5D). These results corroborate the presence of functional human motor circuits driven by corticospinal-like neuronal activity.

### The human corticospinal discovery model reveals potential targets for axon regeneration strategies

**Figure 6:**
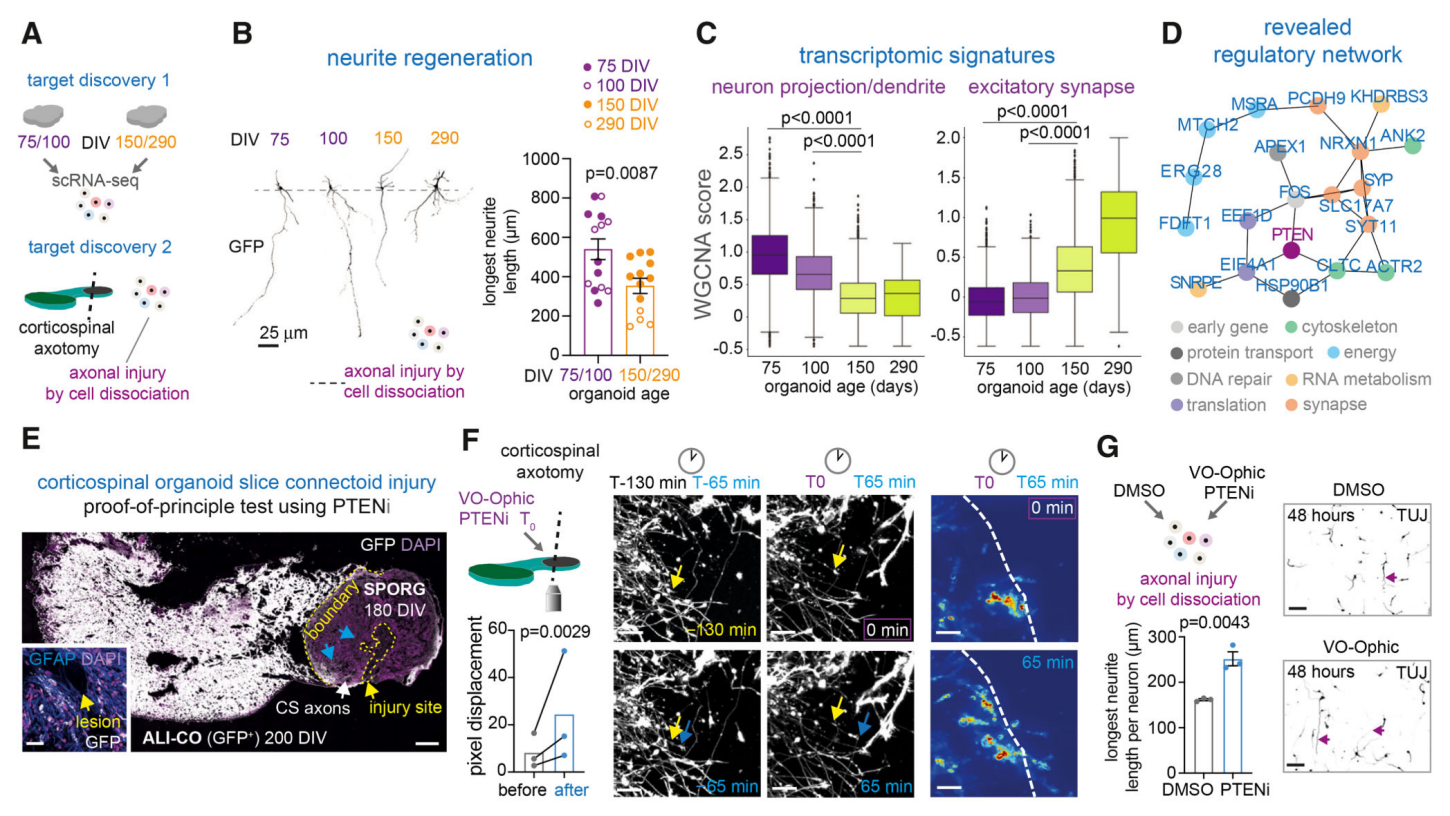
Cortical axon growth restriction is transcriptionally encoded and can be reversed in corticospinal organoid injury (A) Schematic illustrating the target discovery approach using organoid slice and dissociated cell cultures. (B) Representative monochromic immunofluorescence (IF) images (left) of AAV-GFP-transduced GFP^+^ neurons in dissociated cortical organoid cell cultures at different ages. DIV, days *in vitro*. Graph (right) represents mean ± SEM of the longest neurite length of neurons derived from younger (75/100 DIV) and more mature (150/290 DIV) air-liquid interface cortical organoids (ALI-COs). *N* = 14 and 13 neurons, respectively; unpaired two-tailed *t* test with Welch’s correction. (C) Graphs demonstrate results from weighted gene co-expression network analysis (WGCNA). Boxes, lines, and whiskers display the quartile, median, and minimum-maximum distribution (without the outliers) of the top differentially expressed module eigengenes between cortical organoids at 75, 100, 150, and 290 DIV, corresponding to the “excitatory synapses” and “axon guidance” Gene Ontology (GO) terms. Two-sided Mann-Whitney-Wilcoxon test with Bonferroni correction. (D) Core regulatory networks constructed from differentially expressed genes between 75/100 DIV and 150/290 DIV cortical organoids, subjected to graph theory-based analysis using the “betweenness” function in Cytoscape. (E) Representative stitched tile-scan IF image of a human corticospinal (CS) motor organoid-slice connectoid assembled from GFP-expressing 200 DIV ALI-CO slices and non-GFP expressing 180 DIV spinal cord organoid (SPORG) slices with DAPI labeling. GFP^+^ CS axons (blue arrows) crossing the CS boundary were transected (yellow arrow) within the SPORG tissue. Inset shows GFP^+^ axons intervened by astroglial GFAP^+^ processes and DAPI-stained nuclei at the injury site. Scale bars: 400 and 25 μm (inset). (F) Schematic (top left) showing the timeline of the 2-photon imaging following CS-like tract injury and VO-Ophic treatment. Graph (bottom left) illustrating the paired plots and the mean of the top 50 pixel displacements of neurite growth cones for each CS organoid per frame. *N* = 3 independent CS organoids (2–4 regions per sample), ratio paired *t* test. Representative 2-photon confocal microscopy images (middle) from live recordings, showing neurite growth cones (arrows) at the injury site before (T-120 to T-60 min) and after (T0 to T60) the addition of the PTEN inhibitor VO-OHpic (500 nM). Heatmaps (right) indicating average gross pixel displacement over a 60-min period before and after the addition of VO-OHpic (red to blue scale for high to low velocity, respectively). Scale bars: 20 μm. (G) Schematic (top left) illustrating the experimental design. Graph shows mean ± SEM longest TUJ^+^ neurite length in cultures treated with either DMSO or VO-Ophic (500 nM) for 48 h after (200 DIV) cortical organoid cell-dissociation-evoked axonal injury. *N* = 3 independent cultures, two-tailed unpaired *t* test. Representative monochromic IF images of cortical organoid-derived TUJ^+^ neurons. Scale bars: 75 μm.

The human corticospinal motor organoid-slice connectoid system was then exploited to identify targets for corticospinal axon regeneration following injury (Figure 6A). First, we addressed whether limitations in human axon regeneration are developmentally encoded. To do so, cortical axonal injuries were inflicted by dissociation of 75, 100, 150, and 290 DIV ALI-COs, representing early- and late-stage neurodevelopment. Neurons were transfected by the GFP plasmid 3 days after plating, followed by cell fixation and immunolabeling for GFP and measurements of the longest neurite length at day 7. Cortical neurons derived from 150 to 290 DIV organoids displayed a significant 34.51% reduction in neurite regeneration compared to their 75 and 100 DIV counterparts (Figure 6B). Next, we tested the timing of transcriptional changes underlying this observation to pinpoint the most relevant developmental regulators of axon extension block. For this, we ran a meta-analysis on our previously generated ALI-CO scRNA-seq database using weighted gene co-expression network analysis (WGCNA). WGCNA corroborated that the correlation between gene networks regulating axon guidance was significantly reduced between 150 and 290 DIV, at the time of prominent synapse formation, reflected by excitatory synaptogenesis-related gene expression (Figure 6C; Data S1). To reveal genes with regulatory potential underlying this finding, specifically in human deep-layer cortical projection neurons, we projected DEGs between 75–100 and 150–290 DIV ALI-COs onto protein interactions, resulting in large networks. The connections were subjected to graph theory-based analyses, in which the core regulatory elements were defined by the top 20 most connected genes, based on their betweenness score (Figure 6D). This central module indicated functional roles in early transcriptional responses (*FOS*), cytoskeletal regulation (*ACTR2, ANK2*, and *CLTC*), protein transport (HSP90B1), metabolic and energy supply function (*ERG28, MTCH2, MSRA*, and *FDFT1*), DNA repair (*APEX1*), RNA metabolism (*KHDRBS3* and *SNRPE*), translation (*EEF1D* and *EIF4A1*), as well as synapse formation and cell adhesion-associated genes (*SYT1, SYP, NRXN1, SLC17A7*, and *PCDH9*). The confidence in the regulatory potential of these genes was also augmented by their strong connections to phosphatase and tensin homolog (PTEN), a gene known to limit axon extension in rodent models. Thus, to validate the feasibility of targeting this newly revealed core regulatory gene network, we first used VO-Ophic, a PTEN inhibitor compound, to benchmark our functionally mature corticospinal organoid-slice connectoid model for examining post-injury regeneration. For this, we used H9-GFP hESC line-derived ALI-COs assembled with non-GFP SPORGs (Figure 6E). This system allowed assessments of growth cone repair after scalpel-evoked transection of GFP^+^ corticospinal axons, creating an SPORG wound, which was confirmed by visualization of morphological changes of astroglial cells through GFAP immunolabeling (Figure 6E). To accurately assess the effect of PTEN inhibition, we used a particle velocity measurement approach to assess growth cone dynamics reflecting axon repair, since suitable reference points are absent in the dynamically changing injured tissue (Figure 6F). This demonstrated a significant 2.96-fold increase in velocity magnitudes measured at the lesion site within 65 min of recording after VO-Ophic treatment, compared to a 65-min period monitored prior to compound administration, reflecting growth cone movements. To verify that our strategy specifically addresses intrinsic neuronal changes limiting axon repair, we tested the extent of VO-Ophic-induced TUJ^+^ axon regeneration in cell dissociation-related neurite injuries^24^ in sparse ALI-CO-derived cultures. We found a 1.56-fold increase in the longest neurite length of neurons treated with VO-Ophic compared to DMSO (Figure 6G). These findings validated the combined use of our 3D organoid neuraxis and 2D cell monolayer assays as a target-discovery strategy for repairing human cortical axons with a developmentally restricted regeneration program.

### Organoid-based targets inform repurposable drug screening platforms that uncover axon repair enhancers

**Figure 7:**
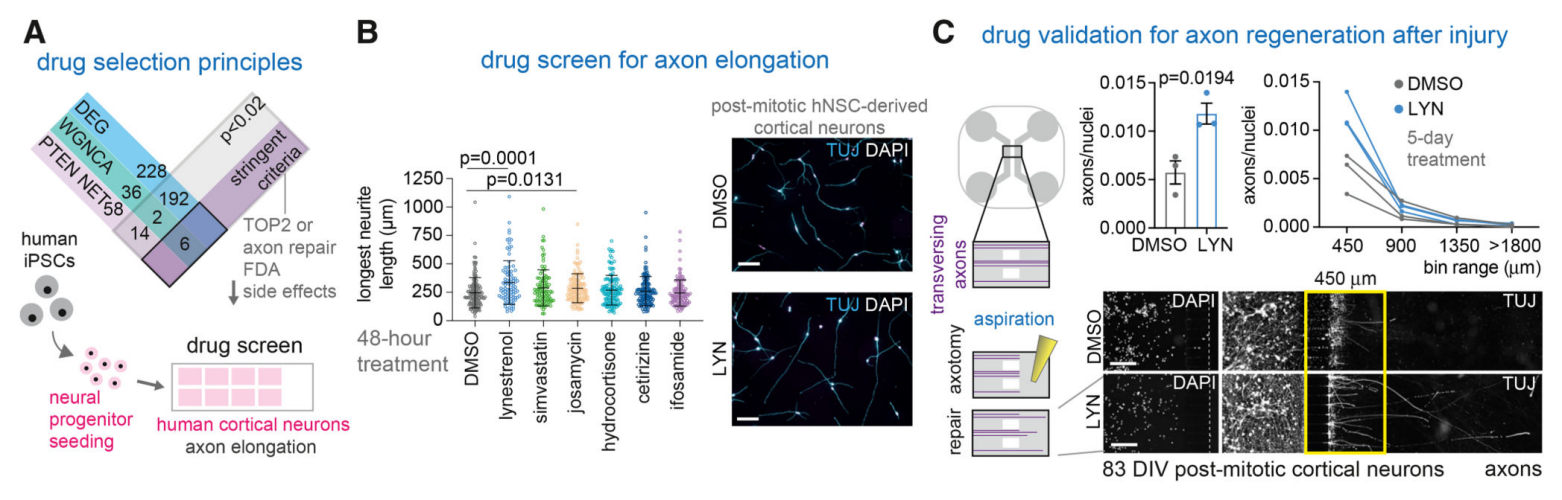
Post-injury cortical axon regrowth is enhanced by drugs targeting developmental growth restrictions (A) Venn diagram illustrating the principles of computational drug selection, based on their predicted potential to reverse gene expression changes in cortical organoids between 75/100 DIV and 150/290 DIV associated with restricted axonal elongation. For drug action predictions, the LINCS, DSigDB, and Proteomics databases were used through Enrichr, based on our input datasets incorporating predicted regulators or axonal elongation: DEGs (differentially expressed genes), WGCNA (weighted gene co-expression network analysis-derived hub genes), PTEN-NET (PTEN-linked network genes within the core regulatory network). (B) Scatterplots (left) showing the mean ± SEM of the longest TUJ^+^ neurite length in human neural stem cell (hNSC)-derived postmitotic cortical neuronal cultures treated with DMSO or 6 FDA-approved drugs for 48 h. *N* = 142, 96, 103, 125, 129, 133, and 121 neurons, respectively for each drug, Kruskal-Wallis test Dunn’s multiple comparisons. Representative immunofluorescence (IF) images (right) of TUJ^+^ hNSC-derived cortical neurons, matured for 21 days post-differentiation and treated by DMSO or lynestrenol (LYN) for 48 h. Scale bars: 75 μm. (C) Schematic (left) of the aspiration-induced axotomy assay using hNSC-derived postmitotic cortical neurons in microfluidic channels. Graphs represent the mean ± SEM (top left) and length distribution (450 μm bins; top right) of the number of regenerated axons per total nuclei of neurons treated with either DMSO or LYN for 5 days after 67 days post-differentiation from hNSCs (83 DIV from the embryonic stem cell state). *N* = 3 postmitotic cortical neuronal cultures in independent microfluidic chambers; unpaired two-tailed *t* test with Welch’s correction. Representative stitched tile-scan IF images illustrating DAPI-stained nuclei and TUJ^+^ neurons (left bottom) in the left-sided chamber and their regenerating TUJ^+^ axons (right bottom) in the right-sided chambers. Scale bars: 150 μm. See also Figures S5 and S6.

We then performed a computational database analysis to identify FDA-approved medications that could broadly target transcriptional changes associated with the failure of axon elongation in deep-layer cortical projection neurons of ALI-COs. We then assembled a target list by identifying DEGs, WGCNA gene hubs, and core regulatory network genes, including PTEN, which served as input for Enrichr-embedded LINCS, HDSigDB, and proteome libraries, resulting in 323 potential effective compounds (Figure 7A; Data S2). Among these, we selected six licensed drugs that were either within the top 2 most significant hits (*p* < 0.02) or those with evidence of axon repair potential and a minimal side effect profile. To prescreen the axon elongation potential of these drugs, we used a readily available rapid mid-throughput cell assay. The platform was assembled using sparsely seeded postmitotic cortical neurons differentiated from H9 hESC-derived human neural stem cells (hNSCs), which displayed immunoreactivity for CTIP2, a deeplayer cortical projection neuron marker, in addition to NeuN and MAP2, neuronal maturity markers (Figures S5A and S5B). We found that lynestrenol was the most potent inducer of axon elongation (Figure 7B). This observation was verified on cortical neurons matured for 67 days post-differentiation state (83 DIV), showing a significant 2.06-fold increase in axon length by lynestrenol following aspiration-evoked injury in microfluidic chambers (Figures 7C and S6A). Our results indicate that discoveries made using the ALI-CO and corticospinal connectoid models have successfully informed drug selection strategies for cortical neuron-based screening platforms, suitable for directly validating the reversal of the intrinsic limitation of axon regrowth.

## Discussion

The timing and causes of the restricted regeneration potential of axons in the human CNS, a major obstacle in brain and spinal cord regeneration, are poorly understood.^8,9^ Using our human ALI-CO and corticospinal motor organoid-slice connectoid models, we revealed that axon guidance and growth machinery of cortical neurons are transcriptionally deregulated at stages representing late fetal development. Examining this early transcriptional shift offered a unique opportunity to identify regulators of this restrictive process in cortical deep-layer projection neurons. By targeting regulatory elements, we show that the pre-programmed limitation of axon elongation can be reversed in an injury paradigm. Building on these findings, our transcriptional target-guided drug-selection strategy paved the way for screening in a scalable neural stem cell-derived cortical neuron-based assay, in which the reversal of intrinsic axon growth restrictions can be directly validated. Ultimately, this study has uncovered the potential of lynestrenol, a repurposable FDA-approved medication, to successfully target the axon-regeneration machinery in human cortical neurons, leading to regrowth. This strategy is plausible to pursue given demonstrations that axons of early neurons with retained neurite growth capacity can transverse even non-permissive environments typical of postnatal or adult spinal cord injuries.^6^

A prerequisite of neural organoid models for studying degenerative or regenerative mechanisms relevant to the human corticospinal motor system is the recapitulation of cell diversity, maturity, and region-specific cellular environments with functional connectivity. There have been challenges in generating complex organoids with distinct spinal cord tissue architecture enriched in motor circuit-forming cells representing a broad ventral as well as rostrocaudal cellular identity.^12,13,25^ We overcame this by optimizing SAG concentrations to mimic SHH gradients for ventralization while caudalizing the tissue using retinoic acid and by supporting spinal MN and IN maintenance by GFs that are normally provided by caudal neuroectodermic and mesodermic elements during development.^26^ Our efforts resulted in several advantages.

We detected wider rostrocaudal cell identities, mirroring not only cervical positions but also a more prominent thoraco-lumbar HOX gene signature than seen for motor assembloids.^12^ This regional representation increased the relevance of our model to conditions leading to neuronal dysfunction across various spinal cord levels. These disorders include traumatic SCI that commonly occurs at cervical and thoracic regions^27^ and also neurodegenerative disorders affecting the spinal cord at multiple levels, such as amyotrophic lateral sclerosis (ALS).^28^ In addition to the wide rostrocaudal representation, the spatial segregation between the cortical and spinal cells while they remain connected by axonal tracts is a practical advantage. This connectoid design enables more accurate investigations of cortical cell-type-specific roles and behaviors without the potential confounding effect of cells from spinal regions, which may otherwise be compromised in directly fused organoid systems.

The spinal organoid slices displayed a CNS-like tissue environment, reflected by highly arborized astroglial and oligodendrocyte processes engaging with neurons and their axons. In particular, our SPORG slice model provides unique advances by resolving ependymal cells with dorsoventral transcriptomic diversity (floor plate versus lateral positions) and by capturing the formation of central canal-like lumen structures lined with ependymal cilia. The 3D arrangement of diverse glial and neuronal populations in SPORGs allows bona fide cell interactions, enabling consideration of non-cell-autonomous processes when examining spinal cord pathologies or repair in neuronal networks.

Additionally, our combined SGE strategy provided incremental benefits over approaches that achieved a broad cell diversity but limited ventral IN phenotypes.^25,29^ The SAG-embedding-based patterning led to the presence of ventral MN phenotypes, and the additional cocktail of GFs increased MN abundance without a major negative impact on IN populations, allowing observations for a longer duration. This methodological step was essential, as our study aimed to examine the decline in axon growth potential of human cortical projection neurons during the critical maturation stages at which they form connections with spinal MNs and INs. Beyond a significant MN enrichment, fine-grained single-cell transcriptomic, morphological, and functional analyses revealed a broad spectrum of mature spinal IN and glial subtypes within the SPORGs.

Regarding the modeling of anterior horn motor circuits, a particular value in our study is the functional validation of MN and IN subtypes, distinguished by their AP characteristics, using single-cell patch-clamp electrophysiological measurements. This was corroborated by their specific transcriptomic signatures and cell marker immunoreactivity profiles, which indicated the abundance of V2a and V2d INs, in particular. These are locomotor pattern generators described in rodents; however, the role of their human counterparts has been challenging to study. Only a few studies reported the generation of 2D monolayer cultures of V2a cells from human iPSCs (hiPSCs)^30^ and their existence in organoid models.^12^ Furthermore, we also detected transcriptional signatures for CSF-contacting neurons, a less well-characterized subtype. Thus, our 3D SPORG model opens new possibilities for exploring their potential role in human neuronal circuits.

A central question regarding modeling human motor circuits is whether cortical neurons in ALI-COs are functionally mature enough to activate the relevant spinal neurons. We have previously provided transcriptomic evidence of deep-layer cortical projection neuron maturation and spontaneous neuronal activity in ALI-COs.^11,19^ Stimulation of cortical neuronal activity also resulted in muscle contractions in a chimeric *in vitro* system comprising human cortical organoids and mouse spinal neuromuscular explants.^11^ In addition, electrical or optogenetic stimulation of cortical organoids within human motor assemblies grown as a single assembloid motor unit led to spinal neuron activation, demonstrating the electrophysiological maturity of corticospinal-like tracts.^12^ In our work, the single-cell patch-clamp studies using the connectoid model indicated that cortical neurons establish both functional monosynaptic and relayed connections onto spinal MNs and INs in SPORGs. In support, direct corticospinal synapses were visualized by the proximity of SYT1^+^/GFP^+^ axon terminals of cortical neuron and HOMER^+^/MAP2^+^ dendrites of ISL1^+^ spinal MNs or INs, including CHX10^+^ V2a cells. Of note, these results mirror the wiring differences between the rodent and the human spinal cord in which corticospinal neurons often directly project onto MNs, a feature exclusively found in certain primates.^31^ Furthermore, the functional maturity of this organoid motor system was demonstrated by the contraction of human myospheres in response to electrical ALI-CO or SPORG stimulation. Overall, these advances justify the use of the human corticospinal motor organoid neuraxis model, an easily accessible connectoid system for conducting mechanistic studies, to uncover the human aspects of corticospinal function and dysfunction.

Multiple transcriptional studies reported that brain organoids follow the developmental milestones of cortical development^19,32–34^ and, as such, are a surrogate model for studying regulators of neuronal differentiation, axon guidance, synaptogenesis, and maturation. We used this as an opportunity to explore if human axon regeneration failure is predetermined at developmental stages represented by ALI-COs at varying ages. This concept has been supported by previous observations in rodents, describing a halt in axon elongation while neurons mature and establish synapses.^35^ Although RNA-seq and chromatin accessibility studies showed that this process is transcriptionally and epigenetically coded,^9,36^ it has been unclear whether this occurs in the developing human CNS and, if so, can be overridden to promote axon repair in injury. We found plausible regulatory candidates through regulatory network analyses of our previously generated datasets representing transcriptional dynamics in developing deep-layer projection neurons in cortical organoids. The fidelity of the network elements in restricting axon elongation was benchmarked by their association with PTEN, a TF shown to control axon elongation during mouse CNS development^37^ and *in vitro* neurite outgrowth of uninjured hESC-derived neuronal progenitors.^38^ The cardinal role of this network was corroborated by the ability of PTEN inhibitor VO-Ophic to reverse axon growth limitations following scalpel and dissociation-evoked axonal injuries in corticospinal organoid-slice connectoids and ALI-CO-derived monolayer cell cultures, respectively. Thus, our work provides direct evidence that both developmental axon elongation and regeneration of human cortical neurons can be reversed, similarly to mouse corticospinal neurons.^39,40^ Crucially, the aforementioned findings shed light on the function of previously unidentified regulatory elements uncovered by our transcriptomic studies. Among more obvious candidates regulating cellular adhesions, cytoskeletal structure, protein translation, and transport, molecules such as the ribonucleoprotein SNRPE, involved in RNA metabolism, and TF APEX1, with DNA repair functions, provide new examples of potential targets. In support, two recent papers report that neuron-specific ribonucleoprotein complexes and DNA repair proteins can regulate axon regeneration of primate retinal ganglion neurons^41^ and mouse dorsal root ganglion cells,^42^ respectively. Overall, our findings confirmed the fidelity of our ALI-CO and organoid-slice connectoid platforms in revealing a network of intrinsic neuronal regulators as targets, which could inform drug-based strategies to promote human axon regeneration after the developmental closure of axon regrowth.

In order to predict the most potent drugs using compound-target interaction databases, we opted to cover a broad set of transcriptional targets instead of single mechanisms identified in our cortical organoid slice-based analyses. This approach helped increase the yield for finding effective strategies in our validation studies. We chose six FDA-approved drugs with the highest potential to promote axon growth, which we then verified in cortical neuron monoculture assays. Lynestrenol, a synthetic progesterone receptor agonist that had been initially used since 1960 for endometriosis, and later as a contraceptive preparation,^43^ turned out to be the most potent enhancer of axon regeneration in our work. Despite its recognized impact on hormonal balance, with a weak androgenic/estrogenic activity and anti-gonadotrophic effects, it has a favorable side effect profile in both women and men.^44^ These features may increase their suitability for potential drug repurposing strategies designed for treating brain or spinal cord trauma. Progesterone has been shown to provide a number of neuroprotective effects in rodent and human CNS and in phase 2 clinical trials in traumatic brain injuries,^45,46^ reflected by more favorable functional scores. While our work supports the rationale for expanding pre-clinical or clinical investigations in CNS injuries using progesterone-related drugs, it provides a platform for broader screens.

Here, we demonstrate that the ALI-CO and organoid-slice connectoid models are highly suitable for discovering targetable molecular signatures underlying the developmentally imprinted restriction of axon elongation. By targeting intrinsic neuronal regulators or multiple transcripts simultaneously, using low- and mid-throughput human organoid- and neuron-based screening assays, we show that limited human cortical axon growth can be reversed by repurposable drugs. We propose that our translational discovery framework, based on integrated 2D/3D platforms, could be widely adopted for mechanistic or drug discovery studies of other neurological diseases affecting the corticospinal motor system, including ALS.^19^

### Limitations of the study

In this study, we employed a single hESC line, given its documented suitability for generating reproducible neural organoid platforms. We chose to use several batches of the same line to ensure consistency in mechanistic studies, thereby eliminating potential line-dependent confounding effects. However, exploiting additional patient-derived cell lines is warranted in studies designed to explore individual differences in repair mechanisms, drug responses, or disease relevance. Moreover, we did not specifically address mechanisms overcoming extrinsic road-blocks to axon regeneration.^3,47^ Our organoid system lacks immune, vascular, and connective tissue elements, which play a major role in creating a non-permissive environment in the adult human spinal cord or brain, blocking axon regeneration and synaptic re-organization. Nonetheless, the model opens new opportunities to examine the direct role of the aforementioned tissue constituents in impeding CNS repair by adding them separately. Indeed, there have been examples supporting these strategies, including combining neural organoids with primary adult human fibroblasts, vascular, and immune cells.^48–50^ However, our findings support the notion that reprogramming the mature axon growth machinery to that observed in newborn neurons might, alone, be sufficient to reinstate regeneration.^5^ This possibility is upheld by the discovery that immature neurons can extend their axons significantly, even in the presence of inhibitory molecules in the injured, non-permissive adult mammalian spinal cord.^6,51,52^ Although our platform revealed a regulatory gene network underlying the inhibition of axon regrowth at advanced developmental maturation stages, which has been successfully targeted to enhance repair, we did not address the specific downstream pathways. Exploring potential links, particularly between the expression of the aforementioned network genes, lynestrenol-induced intracellular events, and known pathways influencing axon growth, including PTEN signaling, could inform the development of new regenerative strategies. Finally, while in our paradigm, the drug-induced axon growth cone motility and regeneration distance are observed only over a brief period, it provides direct relevance to injuries within local cortical circuits or in the cervical spinal cord, in which axons may only need to regrow short distances between segments to project onto spinal motor circuits operating small hand muscle function.^53^ Importantly, further studies are required to investigate other projecting neuron types and the actual integration of injured axons into specific motor circuits, which can build on our complex multiregional platform and observations.

## Star★Methods

### Key Resources Table

**Table:** 

REAGENT or RESOURCE	SOURCE	IDENTIFIER
Antibodies		
Mouse anti-Acetylated Tubulin Table S2	Sigma-Aldrich	Cat# T6793; RRID:AB_477585
Rabbit anti-AQP1	Proteintech	Cat# 20333-1-AP; RRID:AB_10666159
Rabbit anti-ARL13B	Proteintech	Cat# 17711-1-AP; RRID:AB_2060867
Mouse anti-ACTB (beta-actin)	Proteintech	Cat# HRP-60008; RRID:AB_2819183
Goat anti-ChAT	Merck Millipore	Cat# ab144P; RRID:AB_2079751
Mouse anti-CHX10	Santa Cruz	Cat# sc-365519; RRID:AB_10842442
Mouse anti-CNNTB1 (b-catenin)	Thermo Fisher Scientific	Cat# 14-2567-82; RRID:AB_1724004
Mouse anti-EZR (ezrin)	Sigma-Aldrich	Cat# E8897; RRID:AB_476955
Rabbit anti-GATA3	Cell Signaling Technology	Cat# 5852S; RRID:AB_10835690
Mouse anti-GFAP	Sigma-Aldrich	Cat# G6171; RRID:AB_1840893
Goat anti-GFP (biotin labeled)	Abcam	Cat# ab6658; RRID:AB_305631
Rabbit anti-ISL1	Abcam	Cat# ab178400; RRID:AB_2927537
Rabbit anti-HOXA5	Invitrogen PA5-69008	Cat# PA569008; RRID:AB_2689498
Mouse anti-HOXA7	Protein tech	Cat# 67112-1-Ig; RRID:AB_2882416
Rabbit anti-HOXB8	Invitrogen	Cat# PA5-67398; RRID:AB_2662621
Mouse anti-HOXB9	Santa Cruz	Cat# sc-398500; RRID:N/A
Mouse anti-HOXC6	Santa Cruz	Cat# sc-376330; RRID:AB_10990304
Rabbit anti-HOXC8	Invitrogen	Cat# PA5-41629; RRID:AB_2608983
Chicken anti-MAP2	Abcam	Cat# ab5392; RRID:AB_2138153
Rat anti-MBP	Sigma-Aldrich	Cat# MAB386; RRID:AB_94975
Goat anti-NANOG	R&D Systems	Cat# AF1997; RRID:AB_355097
Rabbit anti-Nestin	Sigma-Aldrich	Cat# N5413; RRID:AB_1841032
Mouse anti-neurofilament (SMI312)	BioLegend	Cat# 837904; RRID:AB_2566782
Goat anti-SOX2	R&D Systems	Cat# AF2018; RRID:AB_355110
Mouse anti-SYT1	Synaptic Systems	Cat# 105 011; RRID:AB_887832
Rabbit anti-SYT1	Synaptic Systems	Cat# 105 002; RRID:AB_887830
Mouse anti-OCT3/4	Santa Cruz	Cat# sc-5279; RRID:AB_628051
Mouse anti-TUJ1	Abcam	Cat# ab78078; RRID:AB_2256751
Streptavidin, Alexa Fluor 488 Conjugate	Thermo Fisher Scientific	Cat# S32354; RRID:AB_2315383
Goat anti-chicken Alexa Fluor® 647	Abcam	Cat# ab150175; RRID:AB_2732800
Goat anti-mouse AlexaFluor® 568	Thermo Fisher Scientific	Cat# A-11031; RRID:AB_144696
Goat anti-mouse AlexaFluor® 488	Thermo Fisher Scientific	Cat# A-11029; RRID:AB_2534088
Goat anti-mouse AlexaFluor® 647	Abcam	Cat# ab150119; RRID:AB_2811129
Goat anti-rabbit AlexaFluor® 568	Thermo Fisher Scientific	Cat# A-11036; RRID:AB_10563566
Goat anti-rabbit AlexaFluor® 488	Thermo Fisher Scientific	Cat# A-11008; RRID:AB_143165
Goat anti-rabbit AlexaFluor® 647	Abcam	Cat# ab150083; RRID:AB_2714032
Donkey anti-rabbit AlexaFluor® 405	Abcam	Cat# ab175649; RRID:AB_2715515
Donkey anti-goat DyLight™ 488	Thermo Fisher Scientific	Cat# SA510086; RRID:AB_2556666
Donkey anti-rabbit AlexaFluor® 647	Abcam	Cat# ab150063; RRID:AB_2687541
Donkey anti-mouse AlexaFluor® 488	Abcam	Cat# ab150105; RRID:AB_2732856
Goat anti-rabbit HRP	Thermo Fisher Scientific	Cat# 31462; RRID:AB_228338
Goat anti-mouse HRP	Proteintech	Cat# SA00001-1; RRID:AB_2722565
Bacterial and virus strains		
adeno-associated virus 5 (AAV5) gfaABCID-tdTomato virus	gift from Baljit Khakh, Addgene	Cat# 44332
Biological samples		
Normal Goat Serum	Sigma-Aldrich	Cat# G9023
Donkey Serum	Sigma-Aldrich	Cat# D9663
Chemicals, peptides, and recombinant proteins		
Neurobasal	Thermo Fisher Scientific	Cat# 21103049
DMEM/F12	Thermo Fisher Scientific	Cat# 10565018
B-27 Supplement	Thermo Fisher Scientific	Cat# 17504044
*N*-2 Supplement	Thermo Fisher Scientific	Cat# 17502048
KnockOut™ Serum Replacement	Thermo Fisher Scientific	Cat# 10828010
BrainPhys™ Neuronal Medium/SM1	Stemcell Technologies	Cat# 05792
Y-27632	Tocris	Cat# 1254
CHIR99021	Tocris	Cat# 4423
SB-431542	Tocris	Cat# 1614
Retinoic acid	Abcam	Cat# ab120728
Smoothened Agonist (SAG)	Tocris	Cat# 4366
FGF-2	Peprotech	Cat# 100-18B
BDNF	Peprotech	Cat# 450-02
GDNF	Peprotech	Cat# 450-10
IGF-1 LR3	Peprotech	Cat# 100-11R3
Doxycycline	Sigma-Aldrich	Cat# D5207
LDN-193189	Tocris	Cat# 6053
XAV-939	Tocris	Cat# 3748
DAPT	Tocris	Cat# 2634
VO-OHpic	Tocris	Cat# 3591
Simvastatin	Tocris	Cat# 1965
Cetirizine	Sigma-Aldrich	Cat# 89126-1G-F
Josamycin	Sigma-Aldrich	Cat# 59983
Hydrocortisone 21-hemisuccinate sodium salt	Sigma-Aldrich	Cat# H2270
Ifosfamide	Cayman Chemical	Cat# 17562
Lynenstrenol	Adooq	Cat# A17461
Poly(ethyleneimine) solution	Sigma-Aldrich	Cat# P3143
Dendritic Polyglycerol Amine (dPGA)	DendroTek	Cat# DND400
Geltrex	Thermo Fisher Scientific	Cat# A1413201
Matrigel	Corning	Cat# 354234
TRIzol Reagent	Thermo Fisher Scientific	Cat# 15596026
Tetrahydrofuran	Sigma-Aldrich	Cat# 186562
Histodenz™	Sigma-Aldrich	Cat# D2158
Urea	Sigma-Aldrich	Cat# U5128-5G
TissueTek OCT	Agar Scientific	Cat# AGR1180
Sucrose	Sigma-Aldrich	Cat# S7903
Triton X-	Sigma-Aldrich	Cat# T8787
DAPI	Sigma-Aldrich	Cat# D9542
FluorSave	VWR	Cat# 345789
SlowFade Glass	Thermo Fisher Scientific	Cat# S36917
RIPA Buffer	Sigma-Aldrich	Cat# R0278
Phosphate Inihibitor	Thermo Fisher Scientific	Cat# A32957
Protease Inhibitor	Thermo Fisher Scientific	Cat# 15672129
NuPAGE LDS Sample Buffer	Thermo Fisher Scientific	Cat# NP0007
NuPAGE Novex 4-12% Bis-Tris Gel	Thermo Fisher Scientific	Cat# NP0321
Amersham ™ Hybond ® PVDF Western blotting membranes	GE Healthcare	Cat# 10600023
Dry Milk Powder	Sigma-Aldrich	Cat# 70166
ECL Prime Western Blotting Detection Reagent	GE Healthcare	Cat# RPN2232
WesternBright Sirius Chemiluminescent Detection Kit	Advansta	Cat# K-12043-D20
Hybri-Max™ Dimethyl sulfoxide	Sigma-Aldrich	Cat# D2650
Lipofectamine 2000	Thermo Fisher Scientific	Cat# 11668027
Papain	Worthington	Cat# LK003178
Bovine Serum Albumin	Sigma-Aldrich	Cat# A9418
Sodium Alginate	Sigma-Aldrich	Cat# W201502
Calcium Chloride	Sigma-Aldrich	Cat# C1016
Critical commercial assays		
STEMdiff™ Cerebral Organoid Kit	Stem Cell Technologies	Cat# 08570
MycoAlert® Mycoplasma Detection Kit	Lonza	Cat# LT07-318
Direct-Zol RNA Microprep kit	Zymogen Research	Cat# R2060
SsoAdvanced™ Universal SYBR® Green Supermix	Bio-Rad	Cat# 1725270
iScript cDNA Synthesis Kit	Bio-Rad	Cat# 1708890
Chromium Next GEM Single Cell 3′ GEM, Library & Gel Bead Kit v3.1	10X Genomics	Cat# 1000269
Deposited data		
SPORG DIV100 scRNA-seq	This paper	GEO: GSE285558
Original western blots	This paper	Mendeley Data: https://doi.org/10.17632/zcvnhkjty3.1
Experimental models: Cell lines		
H9 (WA09) hESC line	WiCell	Cat# WAe009-A; RRID:CVCL_9773
ioSkeletal myocytes	bit.bio	Cat# io1002
Oligonucleotides		
Primers Table S1	Sigma-Aldrich	Sequences in Table S1
Recombinant DNA		
pT2-CAG-fGFP	gift from Madeline Lancaster, Addgene	Cat# 108714
Software and algorithms		
CellProfiler	https://cellprofiler.org	RRID:SCR_007358
Enrichr	https://maayanlab.doud/Enrichr	RRID:SCR_001575
GraphPad Prism	https://www.graphpad.com	RRID:SCR_002798
Igor Pro	https://www.wavemetrics.com	RRID:SCR_000325
ImageJ/FIJI	https://imagej.net/ij	RRID:SCR_003070
Leica LAS X	https://leica-microsystems.com	RRID:SCR_013673
MATLAB	https://www.mathworks.com	RRID:SCR_001622
MC Rack	Multi Channel Systems	RRID:SCR_014955
MC Tools (MC Data Tools)	Multi Channel Systems	RRID:SCR_014580
MEA-NAP	https://github.com/SAND-Lab/MEA-NAP	https://doi.org/10.1016/j.crmeth.2024. 100901
PIVLab V2.56	https://www.pivlab.de	https://doi.org/10.5334/jors.334
RStudio	https://www.posit.co	RRID:SCR_000432
Zeiss ZEN Microscopy Software	https://www.zeiss.com	RRID:SCR_013672

### Experimental Model and Study Participant Details

#### Human embryonic stem cells and myocytes

One female authenticated H9 human embryonic stem cell (hESC) line was obtained from WiCell (WA09, MTA-W0387) and its use was approved by the UK Stem Cell Bank Steering Committee (REF SCSC23-61), adhering to local regulations. hESCs were grown in StemFlex (Thermo Fisher Scientific, A3349401) on Geltrex (Thermo Fisher Scientific, A1413201) coated plates and maintained at 37°C and 5% CO_2_. Passaging was performed when cells reached 70–80% confluence using ReLeSR (Stem Cell Technologies). Before experimental work, cells were validated for their pluripotency by performing immunofluorescence staining for the key markers Oct4 (Santa Cruz, SC-5279), NANOG (R&D Systems, AF1997) and SOX2 (R&D Systems, AF2018). For generating GFP-labelled ALI-COs, the GFP vector was incorporated into the H9 hESC line using the ‘Sleeping Beauty’ transposase system. In brief, 1.25 μg of a farnesylated GFP (pT2-CAG-fGFP; Addgene, 108714) donor vector and transposase vector (pCAGEN-SB100X, Addgene, 34879) were mixed with 1.6 μL of magnetic transfection beads (Oz Biosciences) in 200 μL of StemFlex media. The solution was incubated for 30 min at 37°C to facilitate DNA binding to the beads. After incubation the mixture was added to the cells which were further incubated for another 30 min on a magnetic plate. Cultures were then maintained and GFP positive colonies were picked and further expanded. For 3D myosphere formation, ioSkeletal myocytes (a gift from bit.bio, io1002) deriving from human pluripotent stem cells of a 55-60-year-old Caucasian male (no known diseases) were matured using the company’s protocol (https://www.bit.bio/products). All cells were screened for mycoplasma (Lonza, LT07-318) every 10 passages. Working banks of cells were karyotyped to assure genetic stability and an absence of chromosomal abnormalities. While the sex-specificity of the cell lines employed in this study does not influence our results, lynestrenol (used for axon repair studies) may affect neurons to a different extent in females versus males.

#### Karyotyping

For karyotyping, H9 ESCs were passaged twice after thawing and cultured in Geltrex-coated T25 flasks (Thermo Fisher Scientific, A1413301) using StemFlex medium (Thermo Fisher Scientific, A3349401). When cultures reached 80% confluency with compact colonies, the medium was fully replaced 2 h before harvesting. To arrest cells in metaphase, 10 μg/mL colcemid (Thermo Fisher Scientific, 15210040) was added for 30 min at 37°C. The supernatant was collected in 15 mL Falcon tubes containing 500 μL fetal bovine serum (FBS) (Thermo Fisher Scientific, 10-439-016). Cells were dissociated into a single-cell suspension using 0.05% Trypsin-EDTA (Thermo Scientific, 25300054) for 5 min at 37°C, then transferred back into the supernatant and centrifuged at 150 x g for 5 min. The pellet was resuspended in 4 mL pre-warmed 0.075 M KCl hypotonic solution (KaryoMAX KCL; Thermo Scientific, 10575090) and incubated at 37°C for 15 min. Cells were slowly pre-fixed with cold 3:1 methanol (Honeywell, 32213-2.5L)/acetic acid (Sigma-Aldrich, 695092-100ML) fixative, centrifuged at 150 x g for 5 min, and subjected to two additional rounds of fixation. The final cell suspension was preserved in 1.5 mL fixative and stored in a parafilm-sealed microcentrifuge tube for standard chromosome imaging and karyotyping by the Cell Guidance Systems Genetics Service, Cytogenetics Laboratory, Cambridge.

#### Air-liquid interface cortical organoid (ALI-CO) generation

Cerebral Organoids (COs) were generated using the Cerebral Organoid Kit from Stemcell Technologies (catalog no. 08570). Cells >70% confluence were dissociated with Accutase (Sigma-Aldrich, A6964) and centrifuged. The pellet was resuspended at 120,000 cells/mL in EB formation media with inclusion of 10 μM Y-27632 (Tocris, 1254). Cell solution was seeded into wells of an ultra-low adherence 96-well plate (Corning, 7007) and left to aggregate. On day 3, media was refreshed with EB formation media excluding Y-27632 and left till day 5 where aggregates were switched to NI media. After 48 h aggregates were embedded in 15 μL Matrigel (Corning, CLS354277) for 30 min and transferred to an ultra-low adherence 24-well plate (Corning, CLS3473) in expansion media. On day 10 organoids were triturated several times to remove excess Matrigel and transferred to a 50 mm^2^ dish (Appleton Woods, SC265) in IDM-A. From day 10, organoids were grown on a Celltron Shaker (Infors HT) rotating at 57 rpm. After day 35, Matrigel (1:50) was also added to the culture media. Between day 40–55 ALI-CO slice cultures were prepared as previously described.^11^ In brief, whole organoids were embedded in a 3% agarose solution (Sigma-Aldrich, A9414) and sliced at 300 μm using a Leica VT1000S vibratome. Slices were maintained at the air-liquid-interface on 6-well membrane inserts (Millipore, PICM0RG50).

#### Spinal organoid (SPORG) generation

Stem cells were first dissociated to single cells as previously described and seeded at 12,000 cells per well of an ultralow adherence 96-well plate in N2B27 (50:50 Neurobasal/DMEM:F12, 0.5x B-27 supplement, 0.5x *N*-2 supplement; Thermo Fisher Scientific) media containing 10% Knockout Serum Replacement (Thermo Fisher Scientific, 10828010), 10 μM SB-431542 (Tocris, 1614), 3 μM CHIR99021 (Tocris, 4423), 10 μM Y-27632 and 20 ng/mL FGF-2 (Peprotech, 100-18B). Plates were incubated for 3 days allowing formation of EBs. Media was then changed to N2B27 + 10% KOSR containing 10 μM SB-431542, 200 nM retinoic acid (Abcam, ab120728), and SAG (Tocris, 4366) at a range of concentrations. At day 6, media was aspirated and refreshed with N2B27 + 10% KOSR including retinoic acid (200nM) and SAG at a range of concentrations. Media was refreshed every 3 days until day 15. At day 15 organoids were transferred to 50 mm^2^ dishes and cultured on a shaker in N2B27 + 10% KOSR containing 200 nM retinoic acid and 20 ng/mL FGF-2. Organoids were then maintained from day 20 in IDM+A with media changes every 3–4 days. For growth factor experiments, organoids were switched to IDM+A with the inclusion of specific growth factors (Peprotech). For embedding experiments, organoids were embedded in Matrigel on day 6 and subsequently moved to the shaker at this time. The optimized protocol involved ventralizing organoids with SAG (375 nM), and the addition of growth factors from day 20 onwards. SPORG slice cultures were prepared as COs except slicing was performed between day 20–25 and thickness was reduced to 200 μm. SPORGs were either dissociated for monolayer cultures at 15 DIV or grown further as slice cultures with the addition of growth factors as optimized in the text (BDNF+GDNF+IGF-1, 20 ng/mL concentration for all).

#### Human corticospinal motor organoid slice connectoid generation

The corticospinal organoid connectoid model was generated by utilising a mixed Alginate:Matrigel polymer bridge across between cerebral (ALI-CO) and spinal (SPORG) organoid slices. In brief, a 1% alginate solution (in HBSS) was mixed at a 1:1 ratio with Matrigel. ALI-CO (DIV50-60) and SPORG (DIV30-35) slices were transferred from their original membrane to a fresh membrane and a gel bridge was drawn across adjacent organoids by taking 10 μL of Alginate:Matrigel solution and slowly dispersing from organoid to organoid. Organoids were then incubated at 37°C for 30 min. Media was then removed and replaced with 100 mM CaCl2 solution (in HBSS^−/−^ ; Sigma-Aldrich, C1016). After a 1-min incubation, membranes were washed several times with fresh media before being returned to the incubator for further culture. After 3–5 weeks tracts had grown across the polymer bridge, the connectoids were set for further experiments.

#### Myosphere generation

ioSkeletal myocytes (bit.bio, io1002) were thawed as recommended by the supplier and seeded in a 96-ULA-well plate at 100,000 cells per well. For the first 3 days cells were treated with 1 μg/mL Doxycycline (Sigma-Aldrich, D5207) and 10 μM Y-27632. From day 3 onwards 10 ng/mL IGF-1 and 10 ng/mL FGF-2 were added to the recommended media. Aggregates were fed every 2–3 days and cultured for another 7-10 days to generate myospheres. Once myosphere contractions were visible, while retaining a smooth-edged morphology, they were selected for experiments. For electrophysiological studies in the corticospinal organoid slice-myosphere connectoid model, myospheres were placed adjacent to the SPORG slices or any external tracts originating from them.

#### Cortical neural stem cell and neuron differentiation

Confluent hESCs grown on Geltrex were fed with N2B27 containing 100 nM LDN-193189 (Tocris, 6053), 10 μM SB-431542, and 2μM XAV-939 (Tocris, 3748) for 10 days. On day 11, the neuroepithelium was passaged 1:1 using accutase and seeded in N2B27 with addition of Y-27632. From day 12 to day 16 media consisted of N2B27 with no additional molecules. On day 16 neural stem cells (NSCs) were split 1:6 and DAPT (Tocris, 2634) was added to promote neuronal differentiation as published previously.^16^ Neuronal cultures were maintained in N2B27 with 50:50 media changes every 3 days.

### Method Details

#### Organoid cell dissociation

Organoids were dissociated as previously described.^19^ Slices were first transferred from their membrane into a 10 cm^2^ dish containing PBS (Without Magnesium and Calcium) as a wash step. Washed slices were then added to a C-tube (Miltenyi, 130-093-237) containing 2 mL of Papain solution (Worthington, LK003178). The C-tube was then ran on the gentleMACs Octo Dissociator (Miltenyi, 130-095-937) using supplier settings (ABDK). The resulting cell suspension was transferred to a 15 mL conical tube, centrifuged for 5 min at 1000 rpm and resuspended in PBS. Resuspended cells were then strained through a 70 μm filter (Miltenyi, 130-098-462) and diluted 4-fold before being spun down again at 200 g. For cell-based assays, cells were then resuspended in their respective medium with the inclusion of 10μM Y-27632 and seeded according to the experimental requirements. For scRNA-seq, cells were resuspended at 206 cells per μL in a 0.04 BSA/PBS solution.

#### Cell transfection and infection

For the visualization of neuronal neurites, GFP plasmids were generated as previously described.^54^ In brief, a bicistronic vector (pHRsinUbEm) was used where EGFP is expressed under the SFFV promoter and mEmerald fluorescent protein under the ubiquitin promoter. Dissociated cells were transfected using Lipofectamine 2000 (Thermofisher Scientific) as follows. 7 μL of Lipofectamine was mixed with 150 μL of Neurobasal media and 3 μg DNA was mixed with 150 μL of Neurobasal Media at room temperature (RT) for 5 min. DNA and lipofectamine solutions were mixed for 30 min at RT. Media was removed from dishes and 1 mL of warm fresh media together with the lipofectamine:DNA solution was added for 1 h at 37°C. Fluorescent expression level was observed 24–48 h later. For fine grain astrocyte and oligodendrocyte visualization, virus-mediated transduction was performed in SPORGs, using the adeno-associated virus 5 (AAV5) gfaABC1D-tdTomato virus (Addgene, 44332) at the concentration of 1×10^11^ viral particles per mL of PBS. Using a 5 μL Hamilton Syringe, 0.5–1 μL of viral suspension was injected directly into the SPORGs at the periphery, followed by a 24-h incubation before washes and culture media replacement.

#### RNA extraction and qPCR

For extraction of RNA, 4 organoids were pooled in a 500 μL of TRIzol reagent (Thermo Fisher Scientific, 15596026) inside an M Tube (Miltenyi, 130-093-236) and subsequently ran on a gentleMACs Octo Dissociator (Miltenyi, 130-095-937) with the RNA_01 protocol for homogenization. RNA was purified from TRIzol samples using the column based Direct-Zol RNA Microprep kit (Zymogen Research, R2060) with an on-column DNAse clean-up step. RNA was then converted to cDNA using the iScript cDNA Synthesis Kit (Bio-Rad, 1708890) following manufacturers protocol. qPCR of cDNA was then performed using the SsoAdvanced Universal SYBR Green Supermix (Bio-Rad, 1725270) at the recommended settings on a Bio-Rad CFX96 Real-Time System. The primers used are listed in Table S1.

#### Single-cell RNA sequencing and analysis

Single cell libraries were prepared in accordance with the manufacturer using the Chromium Next GEM Single Cell 3^′^ GEM, Library & Gel Bead Kit v3.1 (10X Genomics, 1000269) with a 10X Chromium Controller (10X Genomics, 1000204). In summary, dissociated organoid cell mixture and kit Mastermix were loaded onto a Chromium Single cell 3^′^ chip (estimated capture of 10,000 cells) to generate a gel beads in emulsion (GEM) mixture. A reverse transcriptase reaction was then carried out to generate a barcoded cDNA library using C1000 Touch Thermal Cycler (Biorad, 12 cycles of amplification). Quality control of samples was performed using a 2100 Bioanalyzer Instrument (Agilent) before further processing. For sequencing, samples were pooled and ran on an Illumina NovaSeq 6000. For downstream analysis, output FASTQ files from sequencing were then aligned to the GRCh38 genome using the ‘counts’ function in CellRanger 6.0.0. This produced a gene expression matrix for each sample, with sample 1 containing 14,250 cells with 2,702 median genes per cell and 22,765 mean reads per cell, sample 2 containing 16,058 cells with 2,377 median genes per cell and 20,945 mean reads per cell, and sample 3 containing 17,332 cells, with 2,032 median genes per cell and 15,525 reads per cell. Across all samples fraction reads were >70% (77.5%, 75.7%, and 72.8% respectively) indicating low ambient RNA in the preparations. The common R-package Seurat (v. 4.0.5) was used for downstream processing. Data were first merged in Seurat using the ‘merge’ function, retaining sample specific identification. The merged dataset had a total of 47,460 cells before any filtering and quality control. To exclude strong outliers, cells with a low number of mapped genes, and potentially stressed cells with high mitochondrial genes, the data were filtered using the ‘subset’ function retaining cells with between 500 and 7000 features, and a mitochondrial content less than 15%. Gene expression was then log normalized using the ‘NormalizeData’ function, and variable genes were then identified on the normalized data using the ‘FindVariableFeatures’ command (nFeatures = 2000). Data were then scaled so that the mean expression across all cells was 0, using the ‘ScaleData’ function. Dimension reduction was then performed using principal component analysis (PCA) with the ‘RunPCA’ command using the identified variable features. An ‘ElbowPlot’ was then ran to determine how many dimensions to retain for clustering. This showed a drop-off in variance after around 15 dimensions. Clustering was performed by first constructing a Shared Nearest Neighbor (SNN) graph using the ‘FindNeighbors’ command on the first 15 dimensions. Following this the ‘FindClusters’ function was ran using the Leiden algorithm and a 0.5 resolution to cluster respective cells. To visualize clusters, Uniform Manifold Approximation and Projection (UMAP) reduction was performed and subsequently plotted using the ‘DimPlot’ command. In first round clustering, two clusters showed high expression of genes related to cell stress and hypoxia (*FTL, HSPA5, BNIP3, VEGFA, MALAT1*) and thus were discarded. The subset data were then rerun using the first 12 dimensions for further clustering (Leiden 0.4 resolution). Cluster identities were annotated manually using the top upregulated genes in each cluster. To explore individual cell types, respective clusters were pulled out using the ‘subset’ function and subject to the above workflow. Weighted Gene Coexpression Network Analysis (WGCNA v.1.70–3) was used to identify potential regulatory modules that significantly change in CTIP2-expressing deep layer projection neurons during cortical organoid development. For this, a previously generated scRNA-seq dataset was subjected to meta-analysis, representing a merged dataset of 75, 100, 150, 290 DIV H9-hESC-derived cortical organoids (Data S1 and S2). The data matrix was subset to the top 3,000 variable genes. A minimum module size of 15 and deep split of 4 were retained, and the resulting modules were projected onto the merged dataset using ‘moduleEigengenes()’. These eigenegene values were plotted for all deep layer neurons and the most significant age-associated difference were selected. Enrichment analysis for the genes within each of these modules was performed using STRING (https://string-db.org) to determine the top differences in biological processes between the organoids analyzed at various timepoints.

#### Organoid-human tissue comparison

Single-cell transcriptional datasets from SPORG and human fetal spinal cord cells were integrated using Seurat v4 based on shared genes. After normalization, the top 3,000 highly variable genes were selected via the Variance Stabilizing Transformation method. Harmony was then applied for data integration, producing a corrected low-dimensional embedding in which SPORG and fetal cells were well mixed, while retaining biologically meaningful differences. The integrated data structure was visualized using UMAP. To assess transcriptional similarity between SPORG and fetal cells, we characterized local transcriptional neighborhoods by constructing K-nearest neighbor (KNN) and shared nearest neighbor (SNN) graphs from the Harmony embeddings. From the SNN graph, we extracted all edges connecting SPORG cells to fetal cells, disregarding edges between cells of the same dataset. For each SPORG cell type, we counted the number of cross-dataset edges connecting to each fetal cell type. These counts were then normalized within each SPORG cell type to calculate the fraction of edges linking to each fetal cell population. This fraction served as a similarity score ranging from 0 to 1, with higher values indicating greater transcriptional affinity to the fetal reference populations. To visualize these relationships, we used a Sankey diagram to illustrate the flow of variance from SPORG cell types to their most transcriptionally similar fetal populations.

#### Tissue clearing

Fixed tissues were washed with Milli-Q ddH_2_0 and incubated in a 50% tetrahydrofuran (Sigma-Aldrich, 186562) solution overnight. After rinsing in Milli-Q ddH_2_0, tissues were then immunostained as the previously described^55^ with some minor changes. Blocking incubation was increased to 3 h, primary incubation to 72 h, and secondary incubation to 48 h with multiple washes with PBS in-between each step (3 × 1 h). Tissues were then mounted in either EZ Clear^56^ solution or SlowFade Glass (Thermo Fisher Scientific, S36917). Cleared tissues were imaged using a Zeiss LSM900 and Leica SP5 confocal (20x, 1048×1048 resolution).

#### Immunohistochemistry

Whole organoids and slice cultures were fixed in 4% PFA at RT for 1–2 h. After being washed with PBS, samples were either then stored at 4°C or transferred to a 30% sucrose (Sigma-Aldrich, S7903) solution and left for 24–48 h. Samples were embedded with TissueTek OCT (Agar Scientific, AGR1180) before stored at −20°C. Tissue blocks were sectioned using a Leica CM3050 cryostat and adhered to SuperFrost coated slides (Thermo Fisher Scientific, J1800AMNZ). For immunohistochemistry of organoid sections, tissue was first equilibriated to RT before being blocked and permeabilized with a solution of 0.3% Triton X- containing 10% NGS/NDS for 1 h at RT. Primary antibody (Table S2) incubation was carried in 0.1% Triton X- containing 3% NGS/NDS for an initial 30 min before being left to further incubate overnight at 4°C. The next day, secondary antibody incubation was performed for 1 h at RT in PBS. Following this, sections were incubated with DAPI in PBS for 10 min at RT before being mounted using FluorSave reagent. For the organoid slice connectoid whole scan series, there was the inclusion of a biotin blocking step utilizing the Endogenous Biotin-blocking Kit (Thermo Fisher Scientific, E21390) following manufacturers recommendations. Imaging of synapses and structural features was performed on a Leica TCS SPE and Leica TCS SP8 confocal. High resolution imaging of the central canals was performed on a Zeiss LSM900 Airyscan confocal. For immunophenotyping of SPORGs at DIV45 the 3DHISTECH PANNORAMIC Confocal slide scanner was used with auto-exposure and auto-focus settings. A custom pipeline was created using CellProfiler (v 4.2.1) to analyze these images, overlaying neuron-class-specific markers with DAPI staining.

#### Immunocytochemistry

Cells on coverslips were washed once with PBS and subsequently fixed with 4% PFA (Thermo Fisher Scientific, 28908) for 12 min. Fixed cells were then washed with PBS three times. For blocking and permeabilization, fixed cells were incubated with 0.3% Triton X-(Sigma-Aldrich, T8787) containing 5% Normal Goat Serum (NGS - Sigma-Aldrich, G9023) or Normal Donkey Serum (NDS – Sigma-Aldrich, D9663) for 1 h. Samples were then incubated with respective primary antibody (Table S2) in 0.1% Triton X- containing 2.5% NGS/NDS for 2 h at room temperature (RT). Secondary antibody incubation was performed for 1 h at RT in PBS. For nuclear labeling, the cells were incubated with DAPI (Sigma-Aldrich, D9542) in PBS for 10 min at RT. Coverslips were mounted on slides using FluorSave (VWR, 345789) reagent. Samples were imaged using a Leica DMI8 microscope.

#### Western blotting

Lysates were prepared by pooling organoids in RIPA buffer (Sigma-Aldrich, R0278) with Phosphatase (Thermo Fisher Scientific, A32957) and Protease inhibitors (Thermo Fisher Scientific, 15672129). Samples were incubated on ice for 1–2 h with frequent agitation until tissue dissolution. Lysates were centrifuged (14,000G) and the supernatant collected. Protein concentration estimation was performed using the Pierce BCA Protein Assay Kit (Thermo Fisher Scientific, 23227). For blotting, clarified lysates were mixed with NuPAGE LDS Sample Buffer (Thermo Fisher Scientific, NP0007) and boiled at 95°C for 5 min. Denatured samples were then ran on a NuPAGE Novex 4–12% Bis-Tris Gel (Thermo Fisher Scientific, NP0321). Gels were transferred to a PVDF membrane (GE Healthcare, 10600023) and blocked in 0.1% Tween 20/TBS (TBST) containing 5% Dry Milk Powder (Sigma-Aldrich, 70166) for 1 h at RT. Primary antibody incubation (Table S2) was performed at 4°C overnight in TBST containing 1% Dry Milk Powder. Following this, the secondary antibody (HRP conjugated) was incubated at RT for 1 h in TBS containing 1% Dry Milk Powder. For detection the membrane was incubated in ECL reagent (GE Healthcare, RPN2232; Advansta, K-12043-D20) for 5 min following manufacturers guidelines and subsequently imaged using UVITEC Alliance chemiluminescence imaging system.

#### Cortical organoid tract stimulations and patch-clamp recordings

Organoids and connectoids were transferred to an immersion-type recording chamber and superfused with artificial cerebrospinal fluid (aCSF) (126 mM NaCl, 3 mM KCl, 26.4 mM NaH_2_CO3, 1.25 mM NaH_2_PO_4_, 2 mM MgSO_4_, 2 mM CaCl_2_, and 10 mM glucose, Ph 7.2 and osmolarity 270–290 mOsm/L), which was continuously infused with carbogen gas (95% O_2_/5% CO_2_), and circulated with a peristaltic pump at 2.5 mL/min. Patch pipettes were made from borosilicate glass capillaries (0.68 mm inner diameter, 1.2 mm outer diameter) (World Precision Instruments) with tip resistances of 4–6 MΩ using a P-97 Flaming/Brown Micropipette Puller (Sutter Instrument). Pipettes were filled with intracellular recording solution (IC) (110 mM potassium gluconate, 4 mM NaCl, 40 mM HEPES, 2 mM ATP-Mg, 0.3 mM GTP, pH 7.2 adjusted with 1 M KOH, and osmolarity to 270 mOsm/L with ddH_2_O) which also contained biocytin in a subset of recordings for immunohistochemical staining. Cells were visualized using infrared differential interference contrast (DIC) microscopy (BX51WI Olympus) with a 40× water-immersion objective. Whole-cell patch-clamp recordings were carried out in current clamp mode and data were collected using an Axon MultiClamp 700B amplifier (Molecular Devices). The membrane potential was adjusted to −70 mV by injecting a small hyper- or depolarizing current. Action potentials were elicited by depolarizing current steps (from 20 to 200 pA in 20 pA increments with a duration of 800 ms). Data were acquired via an ITC-18 A/D interface board (Instrutech, Port Washington, New York, USA), and custom-made acquisition procedures in Igor Pro (WaveMetrics, Lake Oswego, Oregon, USA). Offline analysis was performed with MATLAB, IgorPro software and GraphPad Prism. Excitatory postsynaptic potentials (EPSPs) were evoked in one input pathway by direct current pulses (stimulus duration 50 μs) through a metal stimulation electrode, which was placed on the axons of the cortical organoid innervating the spinal organoid or directly on the spinal organoid. The stimulation intensity was adjusted (100 μA–500 μA) to evoke an EPSP with peak amplitude between 3 and 8 mV at 0.2 Hz for at least 5 min. After completion of recordings, organoids and connectoids were fixed and further processed for immunohistochemistry.

#### Connectoid stimulations and myosphere contraction recording

The corticospinal organoid slice-myosphere connectoids were cultured together until frequent spontaneous contractions could be observed. Phase contrast video recordings (10 frames per second) of myosphere contractions were performed using a Leica DMI8 microscope. A stainless steel electrode (A-M Systems, 57100), connected to a constant current stimulator (Digitimer, DS3) was positioned at the front of the ALI-CO component of the connectoid near exiting tracts or within SPORGs, which was manually stimulated at 10 mA (100 μs) to evoke contractions. Stimulations were performed every 15 s following a 1-min baseline recording. Video footages of myosphere contractions were exported from the Leica LAS X software into lif files and downstream analysis was performed in MATLAB (Mathworks) using the PIVLab plugin (v 2.56; https://www.pivlab.de/) to determine velocity changes. After loading the video in PIVLab, it was run through the package with the FFT window deformation algorithm. Two passes of analysis were used: first pass interrogating 64-pixel windows, and the second 32-pixel windows. Post analysis, vector maps were calibrated using the metadata taken from the original Leica videos and further processed with the ‘Derive Parameters’ function to extract velocity magnitudes.

#### Microelectrode array recordings

The corticospinal organoid slice connectoid was transferred to a 3D microelectrode array (MEA) chip (Multi Channel Systems, 60-3DMEA250/12/100iR-Ti-gr) and submerged in BrainPhys media. The corticospinal organoid position on the MEA was adjusted and then secured with a platinum harp with nylon strings. Data was acquired for 2-min per MEA recording at 25,000 Hz with a MEA2100 System using MC_Rack (Multi Channel Systems). Intermittent voltage stimulation (biphasic pulse, 200 ms duration, 0.33 Hz) was provided via a subset of electrodes selected in MC_Rack. The stimulation amplitude was adjusted for each recording for the minimum stimulation to evoke a response in the SPORG (range ±0.5-2V). Data were exported using MC_Tool (Multi Channel Systems) and converted to MATLAB format using custom scripts in MEA-NAP.^57^ Recordings with spontaneous activity were analyzed using MEA-NAP. Spike detection was performed using a threshold of 4x the median absolute deviation of the filtered voltage signal. The spike time tiling coefficient (STTC) lag of 10 ms was used for functional connectivity. Recordings with evoked activity were processed first by removing the simulation artifacts, prior to spike detection and network analysis, using custom MATLAB scripts. Data visualization (Figures 4I and S4B) were generated using MEA-NAP.

#### 2-photon imaging of axon growth cones

2-photon imaging of injured corticospinal organoid slice connectoids was performed using a LaVision BioTec TriM Scope II. One day prior to imaging, the SPORG compartments of the connectoid were cut with a scalpel on one edge. Following a 16-h incubation, the insert was fixed to an imaging dish with commercially available Blu Tack (Bostik) and the dish filled with slice media ensuring coverage of the connectoid. Imaging was performed inside a temperature and carbon dioxide-controlled chamber. For excitation an Insight Deepsee dual-line laser was used, with a 25× water dipping objective recording a z stack of 100 μm depth (1μm intervals) every 390 s at a 1190×1190 resolution. After a period for equilibration, a baseline recording was performed for 2 h. Following the baseline recording, concentrated PTEN antagonist was added (5 μM in slice media) to achieve a 500 nM final concentration. Imaging was then commenced for a further 3-h for post-treatment effects. Before image analysis, time-lapse recordings were first registered using the 3D drift correction plugin in Fiji using previously published principles.^58^ To decrease the noise throughout the image series, a 3D median filter was applied to the registered images (across 5 × 5 × 3 neighborhoods). The pre-registered and filtered series were then transformed into a maximum intensity projection for downstream analysis. For motion estimation, pixel-pixel displacements between frames were computed using optical flow, based on a previously described algorithm.^59^ This algorithm adopts a multiresolution strategy that allows the detection of large displacements. It also tolerates small variations of pixel intensities between successive frames. Furthermore, the algorithm assumes a dense, piecewise smooth flow field, with discontinuities at the boundaries of objects. An analysis of sensitivity to parameters of the optical flow was performed for the two parameters: α – controlling the smoothness of the displacement field and ρ – the variation of the intensity gradient (α = 80,120,160 and ρ = 2,4). As large motile glial cells could be seen around the injury site, regions of interest (ROIs) were selected to isolate areas of clearly motile growth cones from each sample, with each region containing at least one growth cone. As ROIs had variable area size, the largest 50 displacements were selected for each region as a thresholding method.

#### Neurite outgrowth assay

For assays involving dissociated organoids, the single-cell suspension was seeded at 25,000 cells/cm^2^ on PEI+Geltrex coated plates in N2B27+Y-27632. After 1 h, cells were fed N2B27 media containing respective drug treatment (DMSO, VO-OHpic) and further cultured for an additional 48 h before being fixed. For drug screening, NSC-derived neurons were dissociated at 21 days post DAPT addition using papain (30 min, 37°C). These young neurons were seeded as described above and after 1 h changed to the respective treatments at concentrations indicated in the text. After 48 h cells were fixed for subsequent staining. Fixed cells were immunostained for TUJ1 and the longest neurite per cell measured using the NeuronJ plugin (v. 1.4.3) in ImageJ (NIH).

#### Microfluidic axotomy assay

Microfluidics (Xona, RD150) were sterilized in ethanol and mounted on dried dendritic polyglycerol amine (DendroTek, DND400) precoated dishes (Thermo Fisher Scientific, 150682). Geltrex solution was added to both sides of the chamber and left overnight to coat. To seed the microfluidics, NSC cultures were dissociated using accutase and resuspended at 10,000 cells per μL in N2B27 with Y-27632. Geltrex solution was aspirated completely from both sides of the microfluidic before 10 μL of cell suspension was loaded into one side (somatic compartment). Cells were left for 20 min to adhere before being filled with fresh N2B27. After 2 days media was transitioned to BrainPhys+SM1 (Stem Cell Technologies, 05792) with addition of GDNF, BDNF, and IGF-1 to promote terminal differentiation and maturation. Microfluidics were fed every 2–3 days. After a 67-day maturation period post-NSC differentiation by DAPT, axotomy was performed on 83 DIV post-mitotic cortical neuronal cultures by aspirating from the axonal compartment using vacuum pressure. After several washes and aspirations, geltrex solution was then added to the axonal compartment and incubated for 1 h. After incubation, media was changed in both compartments to BrainPhys with respective drug treatments. Microfluidics were fixed after a 5-day treatment, immunostained for TUJ1 and imaged using a Leica DMI8 microscope.

### Quantification and Statistical Analysis

#### General image processing and analysis

For immunofluorescence and confocal microscopy and WB imaging, camera exposure and parameters were kept the same while obtaining images for each experiment. Unmodified images were used for quantitative analyses using ImageJ (v2.0.0 Fiji) or the CellProfiler Image Analysis Software (v0.7; https://cellprofiler.org/). Cell count automation was verified manually. For illustration purposes, the recommended guidelines were followed. Representative images were minimally and uniformly processed in Fiji or in Adobe Photoshop (v21.0.3) without affecting data presentation according to the recommendations. This included changes in exposure parameters in immunofluorescence panels when clear views were obscured in merged images by interference with strong DAPI staining. For obtaining larger views at high resolution, adjacent fields of view were acquired using tile-scanning and stitched to generate composite images of the entire region of interest. For quantifying WB band densities, chemiluminescence was detected by the Alliance 4.7 CCD image system (UVITEC). Band densities were uniformly normalized to a particular sample within the same blot and to β-actin bands in corresponding blots. For focused illustration, the images were cropped, leaving in all lanes with corresponding β-actin loading controls. For all figure assembly, graphs produced in GraphPad Prism (v10.3.0) and images were embedded in Adobe Illustrator (24.0.3), which was also used for producing schematic illustrations. No third-party illustrations were used.

#### Statistics and reproducibility

Sample identifiers were blinded for the observers. Full details of statistical tests and exact samples are provided in Data S3. Sample sizes using hiPSC lines and organoids were estimated from our previously published experiments.^19,60^ Sample allocations into groups included independent organoid slices, connectoids or hESC-derived monolayer cultures (samples with at least three biological replicates/experiments). Statistical tests were only ran using data from biological replicates of independent organoid slices and cell cultures across organoid slices in cell biological assays or different cell subtypes subjected to whole cell patch-clamping recordings. In cases, where technical repeat data points were plotted in repeated experiments for illustration, statistical analysis was not performed. The GraphPad software (GraphPad Prism v10.0) was used for analyzing distribution and normality, statistical tests and generating graphs. In case of non-normal data distribution, nonparametric tests were used. For comparison of two groups, tests included unpaired or paired two-tailed student *t* tests or Welch tests where equal standard deviation was not assumed for normally distributed data, and Mann-Whitney tests for non-normally distributed plots. For comparison of two or more groups, two-tailed analysis of variance (ANOVA) tests were used for parametric data with Dunnett’s or Tukey’s post hoc tests or Kruskal-Wallis tests with Dunn’s post hoc analyses for non-parametric data. The specific statistical test types with exact sample sizes and *p* values are indicated in the figures and legends. Statistical significance was accepted at *p* < 0.05.

## Supplementary Material

**Supplemental Information**

Supplemental information can be found online at https://doi.org/10.1016/j.celrep.2026.117399.

Data S1. Significant module hub genes obtained using WGCNA, related to Figure 6C.

Data S2.

Data S3.

Document S1. Figures S1–S6 and Tables S1 and S2.

Document S2. Article plus supplemental information.

## Highlights

- Maturing human cortical axons connect with both MNs and INs in optimized spinal organoids
- A developmental transcriptional program limits human cortical axon regrowth after injury
- Targeting the restrictive neuronal program reverses the human cortical axon repair block
- Target-informed repurposable drug screening helps find cortical axon repair enhancers

## In brief

Gibbons et al. present a human corticospinal organoid slice-based connectoid model, with physically separate but functionally coupled cortical and spinal regions. Allowing cell-type-specific observations, the platform uncovers a developmental maturation-dependent decline in human axon regeneration and a transcriptional program intrinsic to cortical projection neurons that, when targeted, promotes repair post-injury.

## Resource Availability

### Lead contact

Requests for further information and resources should be directed to and will be fulfilled by the lead contact, András Lakatos (al291@cam.ac.uk).

### Materials availability

This study did not generate new unique reagents.

### Data and code availability

- The SPORG scRNA-seq data generated in this work were deposited under accession number Gene Expression Omnibus (GEO): GSE285558 and are publicly available. Original western blot images have been deposited at Mendeley Data: https://doi.org/10.17632/zcvnhkjty3.1 and are publicly available. Microscopy data reported in this paper will be shared by the lead contact upon request.
- This paper does not report original code. Software availability is indicated in the text and in the key resources table.
- This paper also analyzes existing, publicly available single-nucleus RNA-seq data derived from human fetal spinal cords, which is accessible under accession number GEO: GSE188516. In addition, the cortical organoid time point-related differential gene expression data used for meta-analysis in this work, a non-used subset of our previously collected dataset, are available in Data S2. For drug action predictions, the LINCS, DSigDB, and Proteomics databases were used through the Enrichr platform, which is freely accessible at https://maayanlab.cloud/Enrichr/. Any additional information required to reanalyze the data reported in this paper is available from the lead contact upon request.
