# Supplementary material for: A human corticospinal organoid-slice connectoid model informs enhancer strategies for post-injury axon regrowth
Source: Cell Rep. Author manuscript; Available in PMC 2026 Jul 24. (PMC7619264; doi:10.1016/j.celrep.2026.117399)
Supplement: Document S1. Figures S1–S6 and Tables S1 and S2. [file EMS216068-supplement-Document_S1__Figures_S1_S6_and_Tables_S1_and_S2_.pdf]

Cell Reports, Volume *45*

Supplemental information

A human corticospinal organoid-slice connectoid model informs enhancer strategies for post-injury axon regrowth

George M. Gibbons, Tanja Fuchsberger, Mai Abdelgawad, Stefano L. Giandomenico, Kornélia Szebényi, Veselina Petrova, Lea M.D. Wenger, Daniel N. Olschewski, Jeremi Chabros, Leila Muresan, Rachael C. Feord, Muhammad Asif, James W. Fawcett, Susanna B. Mierau, Ole Paulsen, Madeline A. Lancaster, and András Lakatos

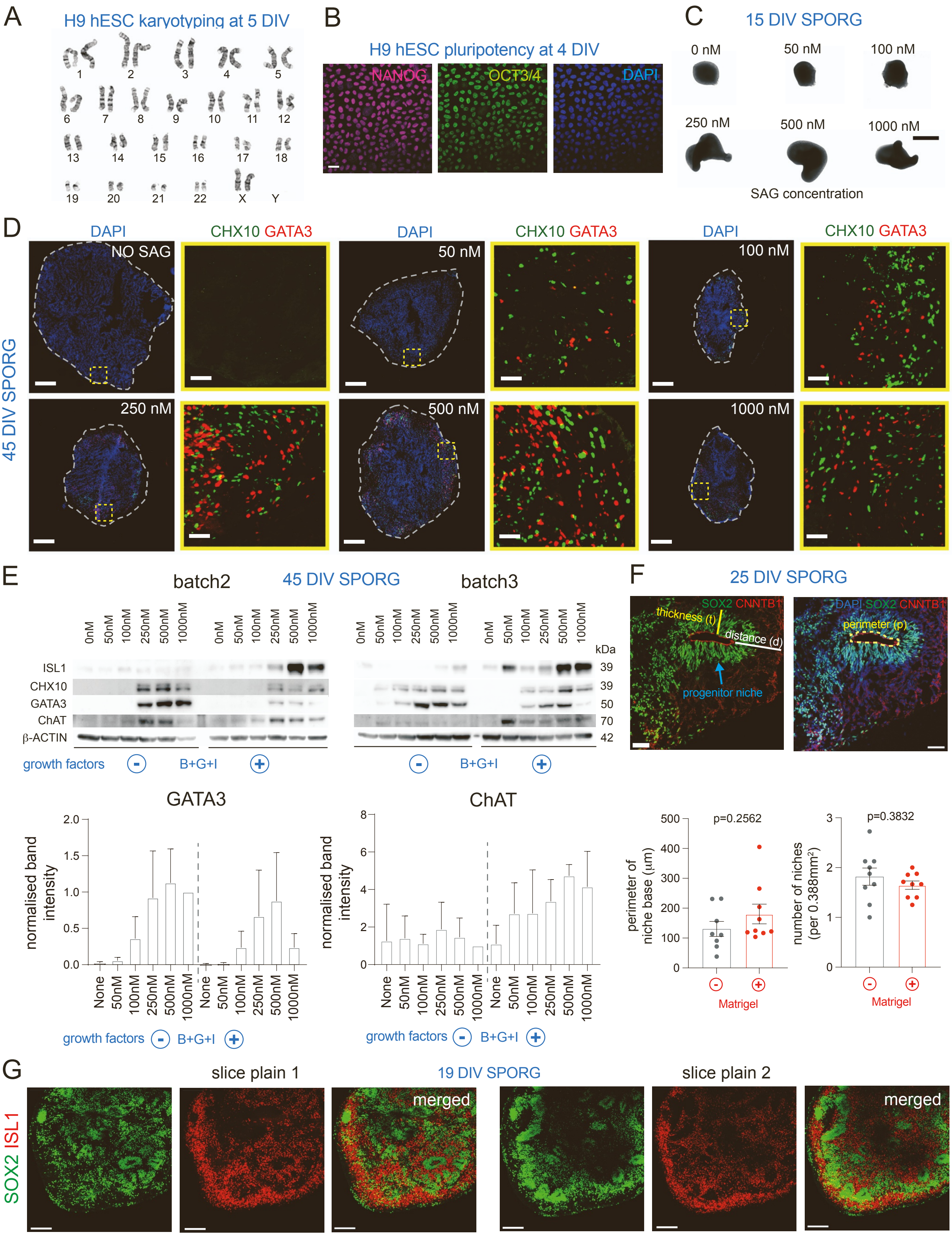

Figure S1. Interneuron and motor neuron abundance is optimized in human embryonic stem cell-derived spinal cord organoids, Related to Figure 1. (**A**) Karyotyping of the H9 human embryonic stem cell line (hESC) line at 5 days-in-vitro (DIV). (**B**) Representative immunofluorescence images of H9 hESCs displaying pluripotency markers at 4 DIV. Scale bar: 20 µm. (**C**) Phase contrast images of spinal cord organoid (SPORG) cultures at 15 DIV, treated with Smoothened Agonist (SAG), a Sonic Hedgehog analogue, at different concentrations. Scale bar: 1000 µm. (**D**) Representative stitched tile-scan confocal microscopy images of DAPI staining and CHX10^{+} V2a and GATA3^{+} V2b interneurons (insets) at 45 DIV in SPORGs treated with SAG at various concentrations. Scale bars: 500 µm, 55 µm (insets). (**E**) Representative western blots for ISL1, CHX10, GATA3, ChAT, and β-ACTIN (top) of 45 DIV SPORG samples either untreated or treated with increasing concentrations of SAG and the BDNF+GDNF+IFG (B+G+I) growth factor (GF) cocktail (20 ng/ml for all). Graphs (bottom) demonstrating band densities normalized to the corresponding β-ACTIN^{+} bands and to ISL1^{+} or CHX10^{+} bands for the 1000 nM SAG-treated SPORG samples in the same blot. N=3 and 4 independent SPORGs for batch 1 and 2, respectively. (**F**) Representative confocal microscopy images (top) of a 500 nM SAG-treated and embedded SPORG at 25 DIV, showing SOX2 immunoreactive (IR) progenitor cell niche with CNNTB1 IR inner/apical zone. Annotations: p=perimeter of the apical zone, t=thickness of progenitor niche, and d=organoid edge-apical zone distance. Scale bar: 50 µm. Graphs (bottom) showing the mean±SEM of perimeter of the apical zone and number of niches in non-embedded and Matrigel-embedded SPORGs at 25 DIV. N=9 independent SPORGs across 3 batches; two-tailed unpaired t-test. (**G**) Single-plain confocal microscopy images of a cleared Matrigel-embedded SPORG at 19 DIV, showing SOX2 IR progenitor niches surrounded by ISL1 IR motor neuron progeny at 65 µm (plain 1) and 100 µm (plain 2) depth within the tissue. Scale bar: 100 µm.

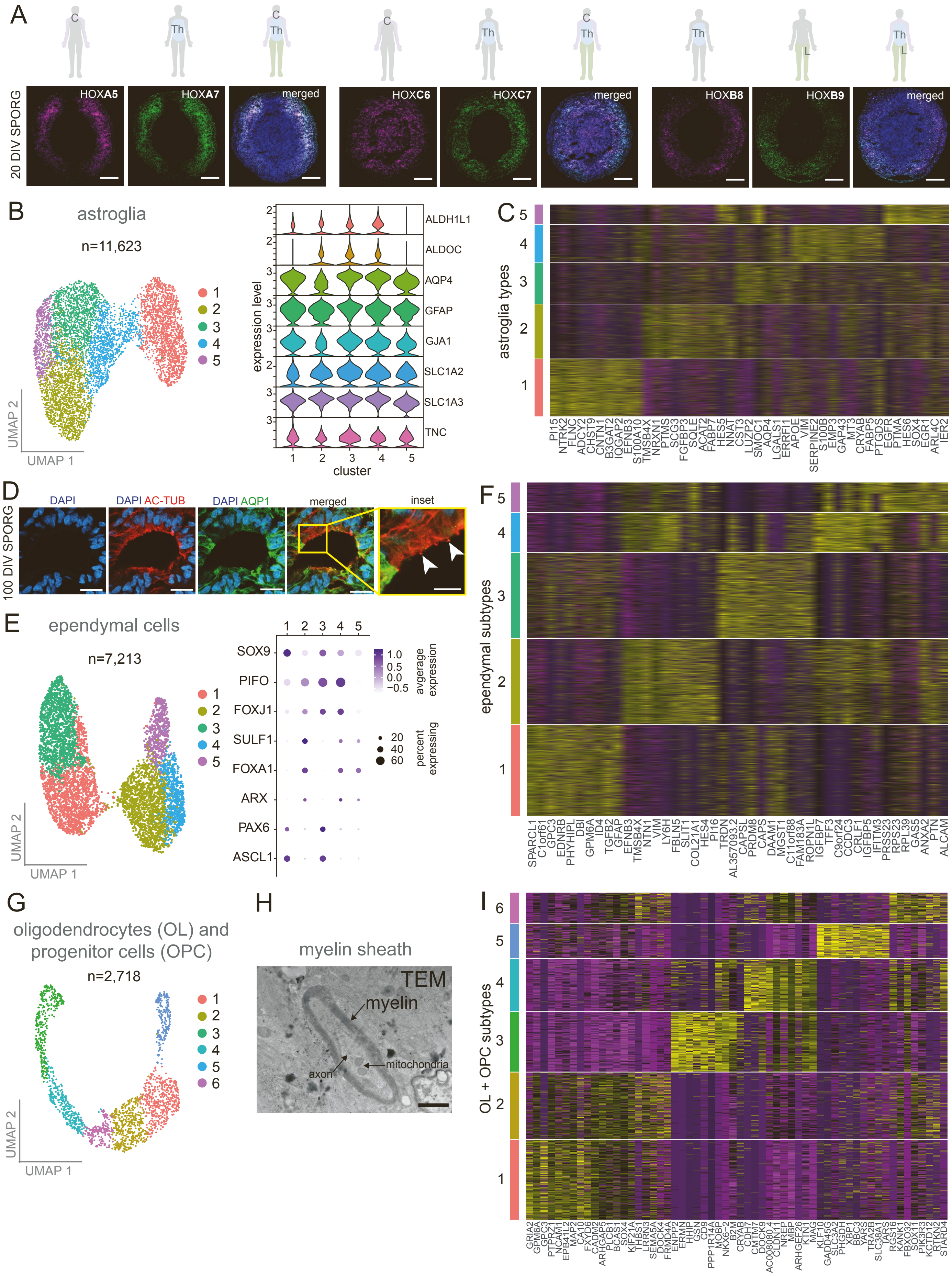

Figure S2. Spinal cord organoids display glial cell type diversity, Related to Figure 2. (**A**) Representative stitched tile-scan immunofluorescence (IF) images of spinal cord organoids (SPORGs) at 20 days-in-vitro (DIV) showing cervical and thoracic HOX protein markers. Scale bar: 300 µm. (**B**) UMAP (left) showing colour-coded clusters determined across astroglial populations (N=11,623). Violin plots (right) demonstrating normalised expression levels of astroglia marker genes per cluster. (**C**) Heatmap illustrating scaled average expression levels of the top 10 differentially expressed genes (DEGs) for each astroglial subcluster (yellow: high expression, magenta/black: low expression). (**D**) Representative confocal microscopy images showing a central canal-like formation by AQP1 and acetylated-tubulin (AC-TUB) immunoreactive cells within a 100 DIV SPORG. Inset shows enriched AC-TUB^{+} cilial protrusions (arrows). Scale bar: 20 µm. (**E**) UMAP showing the subclustered ependymal cell cluster (n=7,213 cells) in 100 DIV SPORGs. Dot plot indicating the relative expression of ependymal marker genes. Colours denote the average scaled expression and dot size indicates the percentage of cells expressing a given gene across the population. (**F**) Heatmap illustrating scaled average expression levels of the top 10 DEGs for each ependymal subcluster. (**G**) UMAP showing the subclustered oligodendrocyte progenitor cell (OPC) and oligodendrocyte (OL) cluster (n=2,718 cells). (**H**) Transmission electron microscopy (TEM) image of a 100 DIV SPORG, illustrating a myelinated axon with mitochondria. Scale bar: 1 µm. (**I**) Heatmap illustrating scaled average expression levels of the top 10 DEGs for each ependymal subcluster (yellow: high expression, magenta/black: low expression).

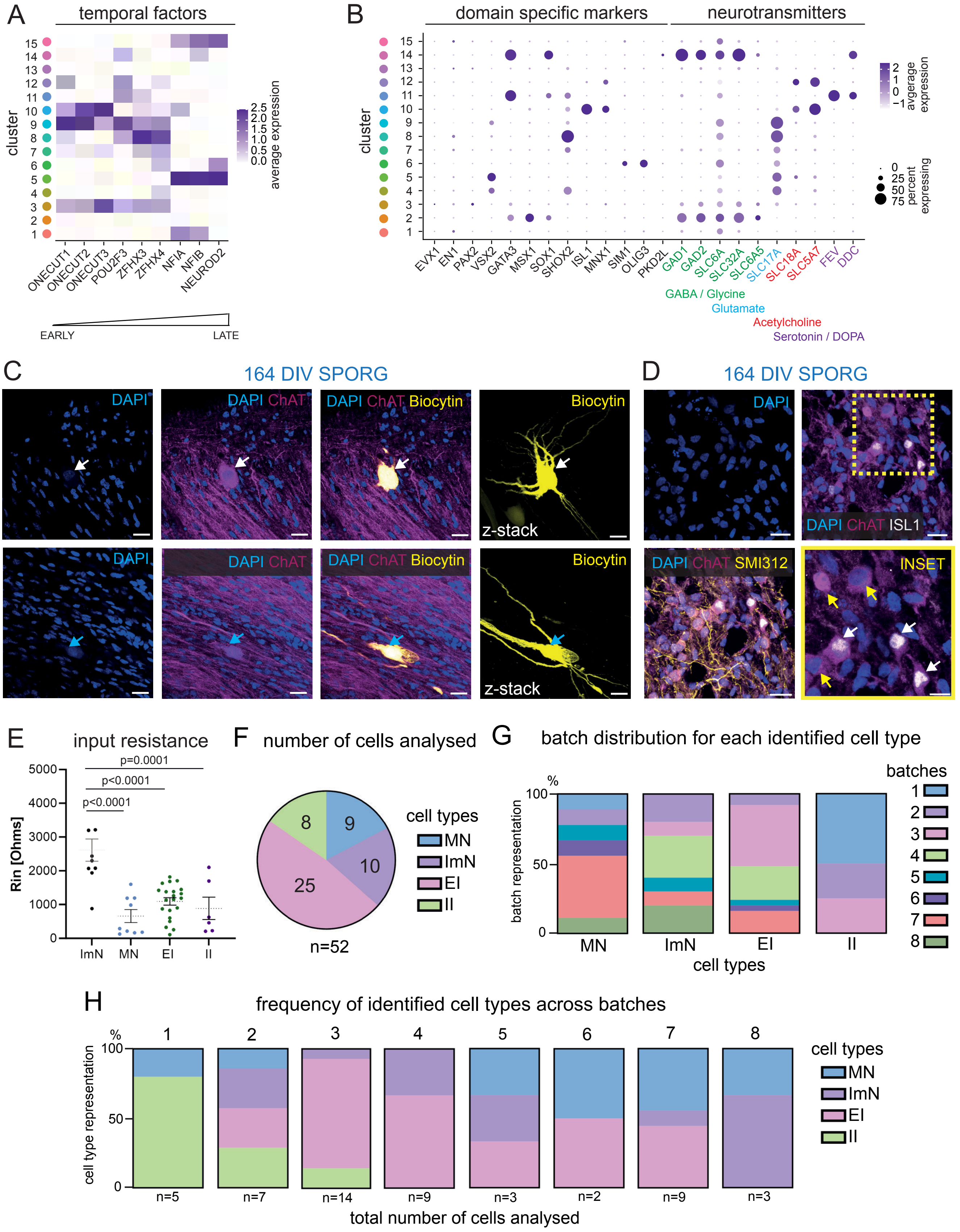

Figure S3. Transcriptional and electrophysiological analyses reveal diverse populations of spinal cord organoid neurons, Related to Figure 3. (**A**) Heatmap demonstrating average gene expression levels of neurogenic genes (colour-coded per cluster), which follows a temporal sequence during development within the 15 neuronal subclusters in spinal cord organoids (SPORGs) at 100 days-in-vitro (DIV). (**B**) Dot plot demonstrating domain-specific marker and neurotrasmitter expression across neuronal populations of three independent SPORGs at 100 DIV. Dot size and colours represent the percentage of cells expressing a particular gene and its scaled mean expression level, respectively (dark magenta: high expression, light magenta: low expression). (**C**) Confocal microscopy images of cleared whole-mount 164 DIV SPORG samples, showing co-localisation (top) of ChAT immunoreactivity within streptavidin labelled biocytin-filled neurons following single-cell patch-clamp recordings. Representative confocal microscopy Z-stack projections (right) showing neurite arborization of biocytin-filled neurons. Scale bar: 25 µm. (**D**) Confocal microscopy images showing DAPI staining and ChAT immunoreactivity in ISL1^{+} motor neurons (white arrows in inset) and ISL1- neurons (yellow arrows in inset) in 100 DIV SPORGs. Scale bar:20 µm and 10 µm (inset). (**E**) Graphs represent relative input resistance (RIN) of various neuronal electrophysiological phenotypes subjected to whole cell-patch clamp recordings in 164 DIV SPORGs. N=10, 9, 21, 6 for MN: motor neurons, ImN: immature neurons, EI: excitatory interneurons, and II: inhibitory interneurons, respectively from 8 independent SPORG batches; one-way ANOVA with Dunnett's posthoc test. (**F**) Parts of whole graph showing the number (n) of cells analysed for each cell type. Colour codes represent motor neurons (MN), immature neurons (ImN), excitatory interneurons (EI), and inhibitory interneurons (II), based on their action potential characteristics. (**G**) Parts of whole graphs demonstrating the percentages of colour-coded organoid batches, in which electrophysiological properties of MNs, imN, EI, and II were represented. (**H**) Parts of whole graphs show the percentages of cell types and the total number of cells analyzed in each batch.

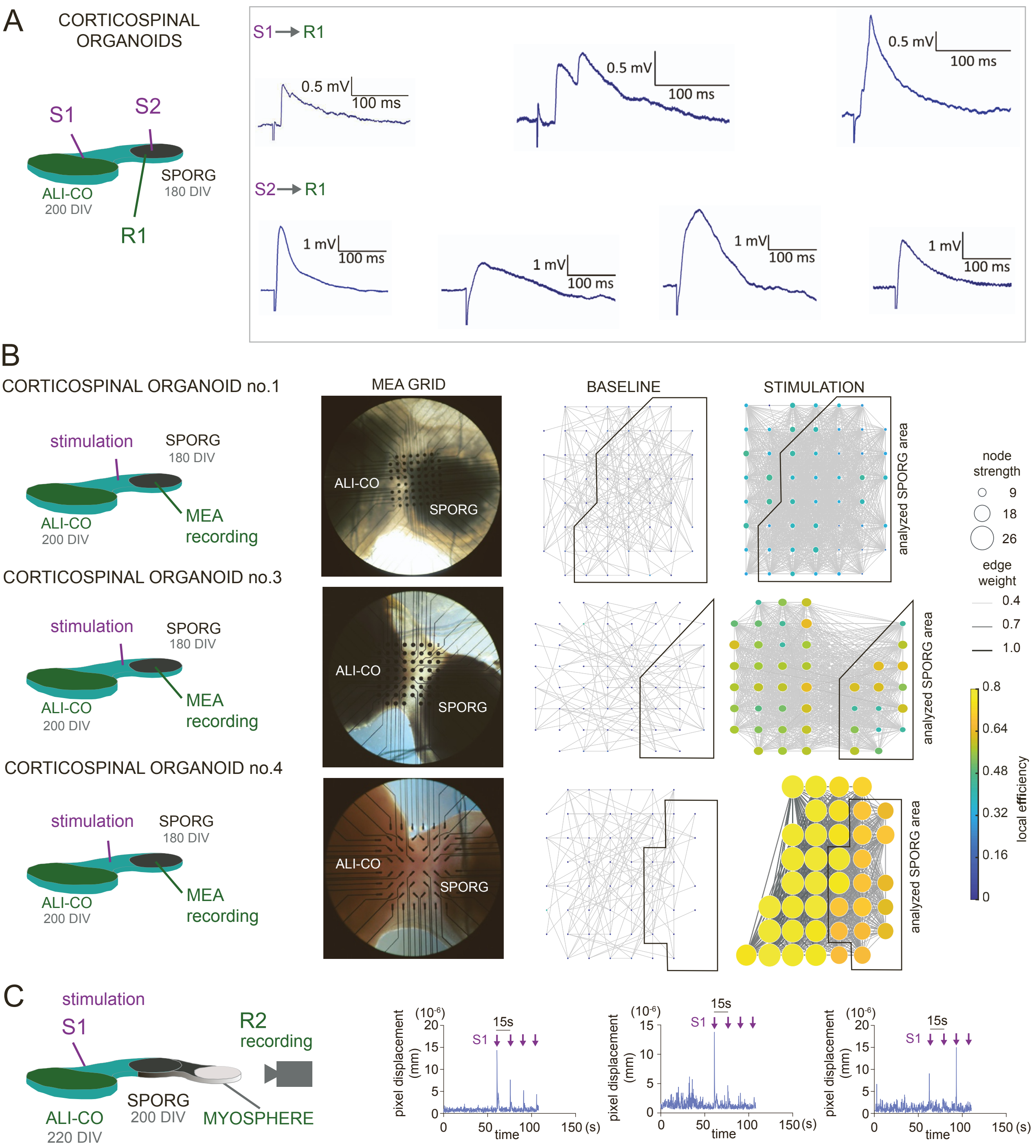

Figure S4. Electrophysiological and video analyses demonstrate corticospinal circuit activity and myosphere contractions, Related to Figures 4 and 5. (**A**) Schematic illustration (left) and recording strategy and representative traces recorded in 180 days-in-vitro (DIV) spinal cord organoid (SPORG) neurons following extracellular stimulation of corticospinal tracts (S1, top) of 200 DIV air-liquid interface cortical organoids (ALI-CO) or local connections (S2, bottom). (**B**) Schematic illustration and phasecontrast images (left) illustrating three independent corticospinal organoids on the microelectrode array (MEA) with overlaying stimulating electrodes (S1) placed on corticospinal tracts of 200 DIV ALI-COs and recording electrodes over 180 DIV SPORGs. Network graphs (right) show functional connectivity within the SPORGs at baseline and during corticospinal stimulation. Node strength (circle size), edge weight (line thickness), and local efficiency (node color) indicate neuronal firing rates, connection strength and domain activity, respectively. Black lines indicate areas of recording electrodes over SPORGs, from which signals had been analyzed. (**C**) Schematic illustration (left) of electrical stimulation of 220 DIV ALI-COs and video recording (R2) of myosphere contractions. Pixel displacement traces (right) recorded from the videos over time with intermittent stimulations (15 seconds apart).

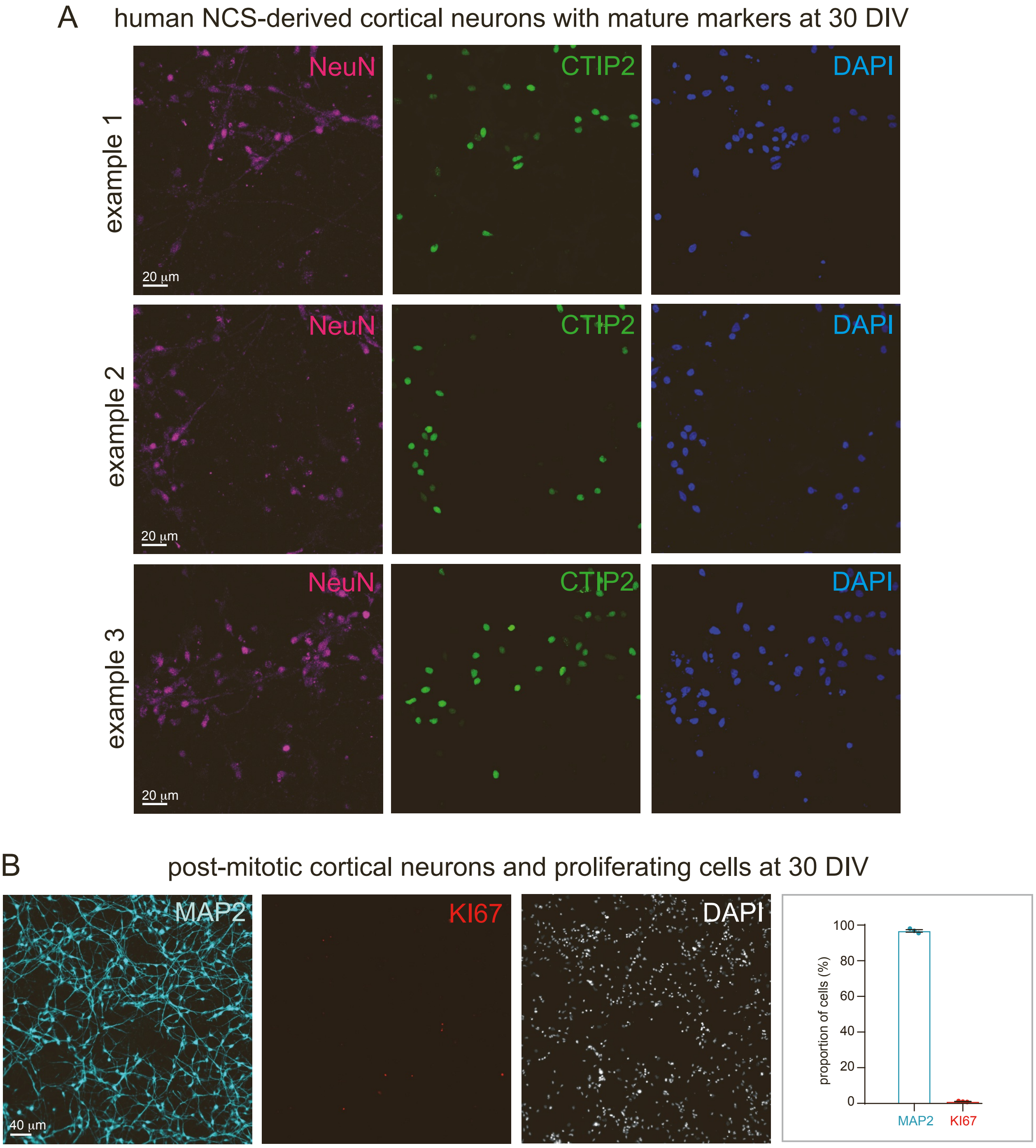

Figure S5. Human neural stem cell-derived cortical neurons are post-mitotic and display maturity markers, Related to Figure 7. (**A**) Representative immunofluorescence (IF) images of human neural stem cell (hNSC)-derived cortical neurons showing immunoreactivity (IR) for mature neuronal marker, NeuN and deep layer cortical projection neuron marker, CTIP2 with DAPI staining at 30 days-in-vitro (DIV; three independent cultures). (**B**) Representative IF images (left) of hNSC-derived postmitotic cortical neurons showing mature neuronal marker, MAP2 IR and a negligible Ki67 IR. Graphs demonstrates the proportions of MAP2 IR and Ki67 hNSC-derived cells at 30 DIV. N=3 independent cortical neuronal cultures. Scale bars: 20 μm for A, and 40 μm for B.

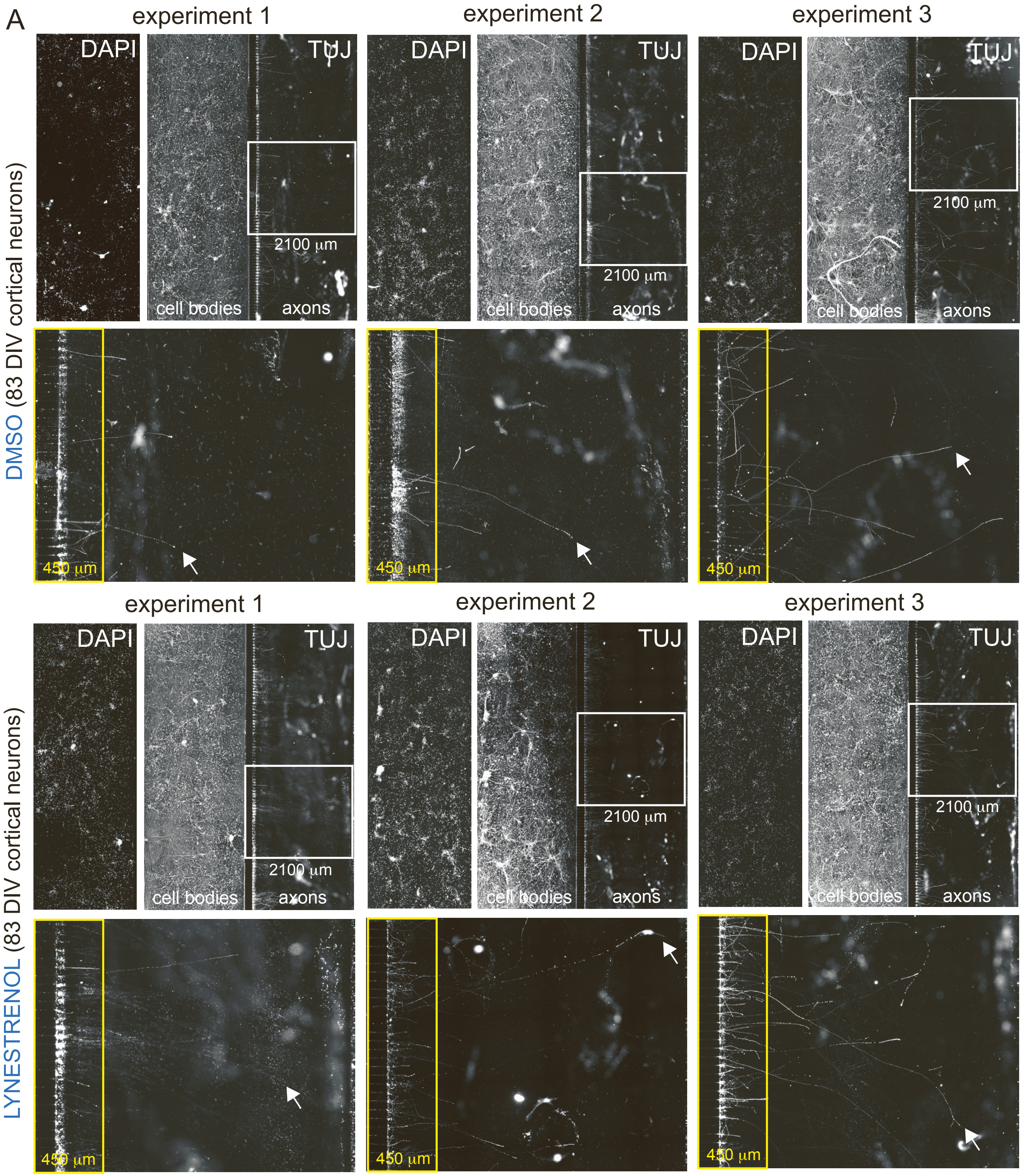

Figure S6. Lynestrenol induces human cortical axon regeneration after aspiration-induced axotomy in microfluidic chambers, Related to Figure 7. (**A**) Stitched tile-scan immunofluorescence images showing human neural stem cell (hNSC)-derived cortical neurons at 83 days-in-vitro (matured for 67 days post-differentiation), which were treated with either DMSO (top panel) or lynestrenol (bottom panels) in microfluidic chambers. The left side of the chamber contains the neuronal cell bodies (DAPI staining) and their initial TUJ immunoreactive axon segments, whearas the right-sided compartment displays axon regrowth 72 hours following aspiration-induced axotomy in three independent cultures for each treatment. Insets show magnified areas defined by the white rectangles, illustrating the number of axons regenerated up to the length of 450 µm (yellow rectangle) and tips of longer axons (arrows).

Target Gene (Forward/Reverse)	Primer sequence (5' -> 3')
DBX1-F	AAG ACC TTC GCC TTT CCC TAC
DBX1-R	CTG CAC GTC GGA GAA GAC TG
DBX2-F	ACT CTA ATT CCA AAG CTC GGA GG
DBX2-R	GGC AAG TTT CTT TCG GTC TGT TT
FOXA2-F	GGA GCA GCT ACT ATG CAG AGC
FOXA2-R	CGT GTT CAT GCC GTT CAT CC
GAPDH-F	AGG GCT GCT TTT AAC TCT GGT
GAPDH-R	CCC CAC TTG ATT TTG GAG GGA
IRX3-F	GAG GGA AAC GCT TAT GGG AGC
IRX3-R	CGC CGT CTA AGT TCT CCA AAT C
NKX2.2-F	CCG GGC CGA GAA AGG TAT G
NKX2.2-R	GTT TGC CGT CCC TGA CCA A
NKX6.1-F	ACA CGA GAC CCA CTT TTT CCG
NKX6.1-R	GCC CCG CCA AGT ATT TTG TT
OLIG2-F	CCA GAG CCC GAT GAC CTT TTT
OLIG2-R	CAC TGC CTC CTA GCT TGT CC
PAX6-F	TGG GCA GGT ATT ACG AGA CTG
PAX6-R	ACT CCC GCT TAT ACT GGG CTA

Table S1. Primer sequences, Related to STAR Methods.

Antibodies/Conjugates	Source/Catalogue Number	Dilution
AC-TUB (acetylated tubulin)	Sigma-Aldrich T6793	1:200 IF
AQP1	Proteintech 20333-1-AP	1:250 IF
ARL13B	Proteintech 17711-1-AP	1:250 IF
ACTB (β-actin)	Proteintech HRP-60008	1:20000 WB
ChAT	Merck Millipore ab144P	1:100 IF, 1:1000 WB
CHX10	Santa Cruz SC-365519	1:250 IF, 1:500 WB
CNNTB1 (β-catenin)	Thermo Fisher Scientific 14-2567-82	1:200 IF
EZR (ezrin)	Sigma-Aldrich E8897	1:200 IF
GATA3	CST 5852S	1:250 IF, 1:1000 WB
GFAP	Sigma-Aldrich G6171	1:1000 IF, 1:1000 WB
GFP	Abcam ab6658	1:500 IF
ISL1	Abcam ab178400	1:250 IF, 1:1000 WB
HOXA5	Invitrogen PA5-69008	1:250 IF
HOXA7	Protein tech 67112-1-Ig	1:250 IF
HOXB8	Invitrogen PA5-67398	1:250 IF
HOXB9	Santa Cruz SC-398500	1:250 IF
HOXC6	Santa Cruz SC-376330	1:250 IF
HOXC8	Invitrogen PA5-41629	1:250 IF
MAP2	Abcam ab5392	1:4000 IF
MBP	Sigma-Aldrich MAB386	1:500 IF
NANOG	R&D Systems AF1997	1:300 IF
Nestin	Sigma-Aldrich N5413	1:500 IF
SMI312 (neurofilament)	BioLegend 837904	1:500 IF
SOX2	R&D Systems AF2018	1:500 IF
SYT1	Synaptic Systems 105 011	1:250 IF
SYT1	Synaptic Systems 105 002	1:250 IF
OCT3/4	Santa Cruz SC-5279	1:300 IF
TUJ	Abcam ab78078	1:1000 IF
Streptavidin, AlexaFluor-488 Conjugate	Thermo Fisher Scientific S32354	1:500 IF
Goat Anti-Chicken Alexa Fluor® 647	Abcam ab150175	1:500 IF
Goat anti-mouse AlexaFluor® 568	Thermo Fisher Scientific A-11031	1:500 IF
Goat anti-mouse AlexaFluor® 488	Thermo Fisher Scientific A-11029	1:500 IF
Goat anti-mouse AlexaFluor® 647	Abcam ab150119	1:500 IF
Goat anti-rabbit AlexaFluor® 568	Thermo Fisher Scientific A-11036	1:500 IF
Goat anti-rabbit AlexaFluor® 488	Thermo Fisher Scientific A-11008	1:500 IF
Goat anti-rabbit AlexaFluor® 647	Abcam ab150083	1:500 IF
Donkey anti-rabbit AlexaFluor® 405	Abcam ab175649	1:500 IF
Donkey anti-goat DyLight™ 488	Thermo Fisher Scientific SA510086	1:500 IF
Donkey anti-rabbit AlexaFluor® 647	Abcam ab150063	1:500 IF
Donkey anti-mouse AlexaFluor® 488	Abcam ab150105	1:500 IF
Goat anti-rabbit HRP	Thermo Fisher Scientific 31462	1:10000 WB
Goat anti-mouse HRP	Proteintech SA00001-1	1:5000 WB

Table S2. Antibody working dilutions, Related to STAR Methods.
